# Transcriptomic Redox Dysregulation in a Rat Model of Metabolic Syndrome-Associated Kidney Injury

**DOI:** 10.3390/antiox14060746

**Published:** 2025-06-17

**Authors:** Chien-Lin Lu, Yi-Yun Wang, Yih-Jeng Tsai, Hsuan-Ting Chen, Ming-Chieh Ma, Wen-Bin Wu

**Affiliations:** 1 School of Medicine, College of Medicine, Fu Jen Catholic University, New Taipei City 242062, Taiwan; 2 Division of Nephrology, Department of Internal Medicine, Fu Jen Catholic University Hospital, Fu Jen Catholic University, New Taipei City 243089, Taiwan; 3 Graduate Institute of Biomedical and Pharmaceutical Science, Fu Jen Catholic University, New Taipei City 242062, Taiwan; 4 Department of Otolaryngology Head and Neck Surgery, Shin Kong Wu Ho-Su Memorial Hospital, Taipei City 111045, Taiwan

**Keywords:** C-C motif chemokine ligand 5, glomerular hyperfiltration, lipid peroxidation, oxidative stress-related gene profiling

## Abstract

Metabolic syndrome (MetS), characterized by obesity, insulin resistance, and dyslipidemia, is a major risk factor for renal injury. Oxidative stress (OxS) plays a pivotal role in its progression; however, the underlying molecular mechanisms are not fully understood. In this study, we established a rat model of MetS using a high-fat diet combined with a single-dose streptozotocin injection in male Wistar rats. MetS rats exhibited systemic OxS, evidenced by elevated circulating levels of free oxygen radicals and decreased antioxidant defense capacity, as well as hypertension, renal lipid peroxidation, glomerular hyperfiltration, and renal tubular injury. Transcriptomic profiling of renal tissue revealed significant downregulation of six OxS-related genes: C-C motif chemokine ligand 5 (CCL5), glutamate-cysteine ligase catalytic subunit, glutathione peroxidase 6, recombination activating gene 2, NAD(P)H: quinone oxidoreductase 1, and selenoprotein P-1. Among these downregulated genes, CCL5 was further confirmed to be repressed at both mRNA and protein levels across intrarenal and systemic compartments. Given its documented functions in immune signaling and redox homeostasis, CCL5 downregulation may contribute to enhanced oxidative damage in MetS-associated renal injury. These findings highlight the role of redox gene dysregulation in the pathogenesis of MetS-related kidney disease and support the potential of CCL5 as a biomarker for oxidative renal injury.

## 1. Introduction

Metabolic syndrome (MetS) has emerged as a major global public health challenge, largely driven by the rising prevalence of obesity and sedentary lifestyles [1]. Defined by a constellation of risk factors—including insulin resistance, hypertension, dyslipidemia, and central obesity—MetS significantly increases the risk of developing type 2 diabetes, cardiovascular disease (CVD), and chronic kidney disease (CKD). Among these comorbidities, kidney involvement has drawn particular attention due to its progressive nature and long-term health implications [2].

Oxidative stress (OxS), defined as an imbalance between reactive oxygen and nitrogen species (ROS/RNS) and the body’s antioxidant defenses, is increasingly recognized as a central contributor to the pathogenesis of MetS. OxS is strongly associated with obesity, insulin resistance, dyslipidemia, and hypertension [3], and is both a cause and consequence of metabolic dysregulation, forming a self-reinforcing cycle. In the kidney, excessive ROS production—primarily from dysfunctional mitochondria and NADPH oxidase activation—disrupts redox signaling and homeostasis, leading to endothelial dysfunction, podocyte damage, glomerulosclerosis, and tubulointerstitial fibrosis [4,5,6,7,8]. These processes promote glomerular hyperfiltration and progressive nephron loss. Systemic OxS further exacerbates lipid peroxidation, inflammation, and vascular injury, accelerating the development of CKD and CVD [9,10,11]. The depletion of antioxidant capacity compounds renal vulnerability to ongoing oxidative insults.

Despite extensive evidence linking OxS to renal injury in MetS, the precise molecular mediators involved in redox regulation and injury progression remain incompletely understood. Recent studies have highlighted the importance of multiple endogenous antioxidant systems—including enzymes involved in glutathione synthesis (such as glutamate–cysteine ligase) [12], peroxide detoxification (such as glutathione peroxidase, Gpx) [13], redox cycling (such as NAD(P)H quinone oxidoreductase 1, NQO1) [14], and selenium transport (such as selenoprotein P-1, SEPP1) [15]—in counteracting ROS and maintaining redox balance. These systems collectively mitigate OxS by catalyzing ROS decomposition, neutralizing free radicals, and restoring redox homeostasis at the cellular and mitochondrial levels [16]. Downregulation of these genes has been associated with impaired glutathione synthesis, redox cycling, and selenium transport, all of which compromise cellular defense against oxidative kidney injury [17,18,19,20]. However, their expression patterns and roles in MetS-associated renal pathology remain underexplored.

C-C motif chemokine ligand 5 (CCL5) is a ligand for chemokine receptor 5 (CCR5) and has traditionally been recognized for its pro-inflammatory role in metabolic and cardiovascular diseases [21,22,23]. Yet, emerging evidence suggests that CCL5 may also exert antioxidative and tissue-protective effects in certain contexts, such as traumatic brain injury, renal fibrosis, and immune-mediated diseases [24,25,26,27]. CCL5 has been shown to activate Gpx1, attenuate transforming growth factor-β1 (TGF-β1) signaling, and modulate macrophage polarization [24]. These findings point to a possible dual role for CCL5 in balancing immune activation and oxidative injury. However, its specific function in MetS-associated renal OxS remains poorly defined.

Given these considerations, the present study aimed to investigate the molecular landscape of OxS-related gene expression in the kidneys of a rat model of MetS induced by a high-fat diet (HFD) and streptozotocin (STZ). We employed transcriptomic screening, histological staining, and multi-level validation to assess systemic and renal redox imbalance. In particular, we explored the expression profile and potential biomarker role of CCL5 in the setting of MetS-related kidney injury.

## 2. Materials and Methods

### 2.1. Animal Model and Experimental Design

Male Wistar rats (7 weeks old) were obtained from Bio-LASCO Taiwan Co., Ltd. (Taipei, Taiwan) and housed under standard conditions (22 ± 2 °C, 55 ± 5% humidity, 12 h light/dark cycle) with ad libitum access to food and water. Following a one-week acclimation period, rats were randomly assigned to either a control group (normal diet) or a MetS group (HFD). The control diet (2018S Teklad Global 18% Protein Rodent Diet, Envigo, Indianapolis, IN, USA) contained 18.4% protein, 6.0% fat, and 44.2% carbohydrate by weight. The HFD (DIO Rodent Purified Diet with 60% Energy from Fat–Blue 58Y1, Test Diet, St. Louis, MO, USA) contained 22.6% protein, 35.1% fat, and 25.9% carbohydrate by weight. Both diets were administered ad libitum throughout the experimental period.

After three weeks on the HFD, rats in the MetS group received a single intraperitoneal injection of freshly prepared streptozotocin (STZ; 35 mg/kg; Sigma-Aldrich, St. Louis, MO, USA) dissolved in 0.1 M sodium citrate buffer (pH 4.5) to induce partial β-cell dysfunction and reduce insulin production. Control rats received an equivalent volume of vehicle (sodium citrate buffer). All animals were fasted overnight prior to injection. The fasting blood glucose (FBG) levels were measured at 1 day before STZ injection and in every week after STZ injection using a glucometer (Accu-Chek Performa, Roche Diagnostics GmbH, Mannheim, Germany). Within this MetS model, hyperglycemia was defined as any fasting blood glucose level markedly surpassing that of the control group, consistent with the glycemic profile characteristic of prediabetic or insulin-resistant states.

**Figure 1:**
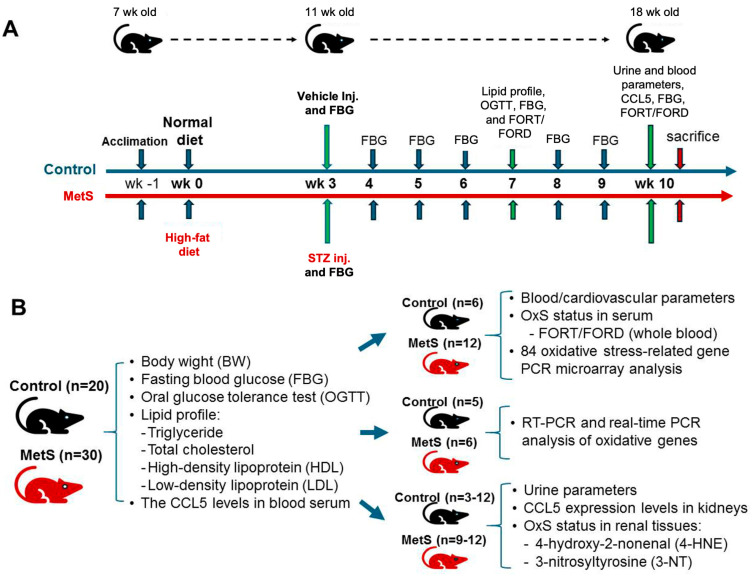
Experimental protocol and sample allocation in metabolic syndrome (MetS) rat model. (**A**) The experimental protocol for MetS induction in Wistar rats is illustrated for 11 weeks. After one week of acclimation, rats were randomly assigned to either the control group (blue timeline) or the MetS group (orange timeline) starting at 8 weeks of age (week 0) fed with normal or high-fat diet (HFD), respectively. At week 3, the MetS group received a single intraperitoneal injection of streptozotocin (STZ) to induce insulin deficiency, while the control group received a vehicle. Fasting blood glucose (FBG) was measured weekly from week 3 to week 10. Metabolic and oxidative stress (OxS) assessments, including lipid profile testing, an oral glucose tolerance test (OGTT), and free oxygen radicals test (FORT)/free oxygen radicals defense (FORD) assays, were conducted at week 7. At week 10, final blood and urine samples were collected for CCL5 measurements, FBG evaluation, OxS markers, and renal oxidative injury analysis, followed by euthanasia. (**B**) The sample distribution for different analyses is summarized. All 20 control rats and 30 MetS rats underwent assessments of body weight (BW), FBG, lipid profiles (TG, triglyceride; TC, total cholesterol; HDL, high-density lipoprotein; LDL, low-density lipoprotein), and serum C-C motif chemokine ligand 5 (CCL5) levels. Subgroups were allocated for specific analyses: blood and cardiovascular parameters, OxS and redox gene expression profiling, renal CCL5 expression analysis by RT-PCR and qPCR, urine parameters, and renal oxidative injury assessments for 4-hydroxy-2-nonenal(4-HNE) and 3-nitrotyrosine (3-NT).

Throughout the 10-week experimental period, body weight was recorded weekly along with water intake. Metabolic and physiological assessments, including lipid profile testing, oral glucose tolerance test (OGTT), and OxS evaluation (free oxygen radicals test (FORT) and the free oxygen radicals defense (FORD) assays), were conducted at week 7. Final blood and urine samples were collected at week 10 before euthanasia to evaluate blood FORT/FORD and CCL5 levels, renal injury markers, and renal function. At the end of the study, animals were sacrificed under deep anesthesia with sodium pentobarbital (60 mg/kg, intraperitoneally) and euthanized for tissue collection. The experimental time points for different treatments and assays are summarized in Figure 1A.

**Figure 2:**
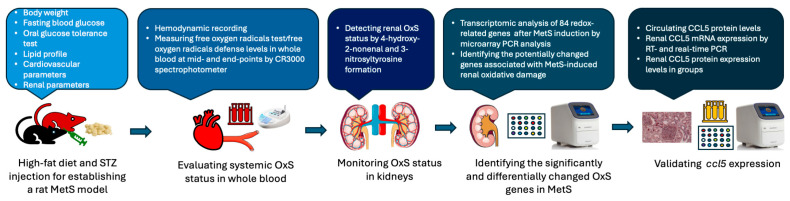
Schematic workflow of the experimental design and analysis pipeline. Metabolic syndrome (MetS) was established in rats using a high-fat diet combined with streptozotocin (STZ) injection. Systemic oxidative stress (OxS) was assessed via the free oxygen radicals test and the free oxygen radicals defense assay performed on whole blood samples. Renal OxS was evaluated by immunohistochemical staining for 4-hydroxynonenal and 3-nitrotyrosine. Transcriptomic profiling of 84 redox-related genes was conducted using a PCR array to identify differentially expressed targets. CCL5 was subsequently validated by various PCR assays in kidney tissues, and protein expression was measured in both serum and renal tissues.

The experimental protocol was approved by the Institutional Animal Care and Use Committee of Fu Jen Catholic University (approval no. A10773). A total of 20 control and 30 MetS rats were enrolled. Specific sample sizes for each analysis are indicated in Figure 1B. The overall experimental and analytical workflow—including MetS induction, systemic and renal OxS assessments, transcriptomic profiling, and candidate gene validation—is presented in Figure 2.

### 2.2. Oral Glucose Tolerance Test (OGTT)

The OGTT was performed at week 7, 4 weeks after the STZ injection. After a 12 h overnight fast, rats received an oral glucose load (1.5 g/kg body weight), and blood glucose levels were measured at 0, 30, 60, 90, 120, and 150 min using a Bayer Contour Plus Glucose Meter (Bayer AG, Leverkusen, Germany).

### 2.3. Blood and Urine Sample Collection

At week 7 and week 10, blood and urine samples were collected for biochemical and OxS analyses. At both time points, whole blood was drawn from either the tail vein (week 7) or the abdominal aorta under anesthesia (week 10). Blood collected into heparinized tubes was centrifuged at 620× *g* for 10 min to separate plasma, which was subsequently used for lipid profile analysis. In parallel, a separate aliquot of fresh heparinized whole blood (not centrifuged) was directly subjected to OxS assessment using the FORT and the FORD assays (see Section 2.6).

Additional non-heparinized blood was allowed to clot at room temperature and then centrifuged (620× *g*, 10 min) for serum collection, which was used for CCL5 ELISA analysis. At week 10, urine was collected over a 24 h period using metabolic cages. Urinary protein concentration was measured using a colorimetric assay kit (Abcam, Cambridge, UK), and lactate dehydrogenase (LDH) activity was determined using the LDH Activity Assay Kit (Abcam, Cambridge, UK).

### 2.4. Plasma Biochemistry Analysis

Plasma samples collected at week 7 were sent to Accuspeedy Medical Laboratory (Tainan, Taiwan) for lipid profile analysis, including total cholesterol, high-density lipoprotein (HDL), low-density lipoprotein (LDL), and triglycerides (TGs). This approach was adopted due to the lack of clinical-grade lipid analyzers in our institutional laboratory. All analyses were performed under standardized conditions by a certified diagnostic provider to ensure accuracy and reliability.

Plasma creatinine concentrations were assessed using a commercial Creatinine Assay Kit (Abcam, Cambridge, UK). Renal function was evaluated by calculating creatinine clearance (CrCl) based on the following formula: CrCl (mL/min) = [Urine creatinine (mg/dL) × Urine volume (mL/1440)]/Plasma creatinine (mg/dL). Urine creatinine levels and 24 h urine volume were obtained from the week 10 metabolic cage collections.

### 2.5. Hemodynamic Parameters Assessment

Hemodynamic measurements were performed at week 10 under anesthesia by intraperitoneal injection of sodium pentobarbital (60 mg/kg). The left femoral artery was cannulated using a polyethylene catheter (PE-50) inserted to a depth of approximately 1 cm and filled with heparinized saline to prevent clotting. Arterial blood pressure and heart rate were recorded via a pre-calibrated pressure transducer (SS13L) connected to the MP-30 data acquisition system (BIOPAC Systems, Goleta, CA, USA). Following a subcostal incision to expose the kidney, a transonic perivascular flowmeter (TS420; Transonic Systems Inc., Ithaca, NY, USA) was placed on the left renal artery to continuously monitor renal blood flow. For each rat, at least three consecutive readings were recorded and averaged to ensure accuracy.

### 2.6. Oxidative Stress Assessment

OxS was evaluated at two distinct time points—week 7 (midpoint of model development) and week 10 (end point before sacrifice)—to monitor the progression of systemic redox imbalance in MetS. In this analysis, 20–50 μL of freshly collected heparinized whole blood was obtained from the tail vein as described above and immediately analyzed using a CR3000 spectrophotometer (Callegari S.R.L., Parma, Italy) [28]. Two standardized assays were employed: FORT and FORD. FORT quantifies circulating hydroperoxides (ROOH), providing a measure of oxidative burden through lipid peroxidation and other ROS-mediated damage. Results are expressed as millimoles of hydrogen peroxide equivalents per liter (mmol H_2_O_2_ Eq/L), using H_2_O_2_ as the standard calibration substance. The FORD assay evaluates total antioxidant capacity based on the sample’s ability to reduce stable preformed colored radicals. Results are expressed as millimoles of Trolox equivalents per liter (mmol Trolox Eq/L). Trolox is a water-soluble analog of vitamin E used as the calibration standard in the FORD assay [28]. Both assays were performed according to the manufacturer’s instructions, and their absorbance was set at 505 nm.

### 2.7. Renal Oxidative Gene Expression Profiling

At week 10, after hemodynamic measurements and blood sampling for oxidative status assessment, as described above, the kidneys were promptly harvested from six control and twelve MetS rats for OxS-related gene expression analysis. Renal tissues were rapidly excised, rinsed in cold phosphate-buffered saline (PBS), and snap-frozen in liquid nitrogen for RNA extraction. Total RNA was isolated using the RNeasy Plus Universal Mini Kit (Qiagen, Hilden, Germany) in accordance with the manufacturer’s instructions. RNA purity and integrity were assessed spectrophotometrically, with A260/A280 ratios between 1.9 and 2.1 considered acceptable. Complementary DNA (cDNA) was synthesized using the iScript cDNA Synthesis Kit (Bio-Rad Laboratories, Inc., Hercules, CA, USA), and OxS-related gene expression was quantified using the RT^2^ Profiler™ PCR Array Rat Oxidative Stress (Qiagen, Product #330231), which includes 84 genes involved in redox regulation, antioxidant defense, and oxidative damage response. This panel, which includes genes associated with ROS metabolism, redox-sensitive signaling, oxidative damage response, antioxidant defense, and related transcriptional regulators, is widely used in OxS research and offers a comprehensive screening of genes known to play pivotal roles in redox homeostasis and cellular oxidative injury. qPCR was conducted using RT^2^ SYBR Green Fluor qPCR MasterMix (Qiagen) on an Applied Biosystems 7300 Real-Time PCR System (Foster City, CA, USA). Glyceraldehyde-3-phosphate dehydrogenase (GAPDH) served as the housekeeping gene. Differential expression was calculated using the 2^−ΔΔCt^ method, and genes showing more than a twofold change with *p* < 0.05 were considered statistically significant.

### 2.8. Renal Immunohistochemistry

After euthanasia, kidneys from six control and twelve MetS rats were collected, rinsed in PBS, weighed, and fixed in 4% paraformaldehyde, followed by paraffin embedding. Paraffin blocks were sectioned into 4 μm thick slices using a microtome. After deparaffinization and rehydration in a graded ethanol series, antigen retrieval was carried out at 95 °C for 20 min using citrate buffer (pH 6.0). Slides were blocked with 10% fetal bovine serum (FBS) and incubated overnight at 4 °C with primary antibodies against CCL5 (sc-365826, dilution 1:50; Santa Cruz Biotechnology, Dallas, TX, USA), 4-hydroxynonenal (4-HNE) (MAB3249, dilution 1:250; R&D Systems, Minneapolis, MN, USA), and 3-nitrotyrosine (3-NT) (sc-32757, dilution 1:150; Santa Cruz Biotechnology). Antibody conditions were optimized beforehand to ensure specificity and consistent signal intensity. Quantitative image analysis was performed using Invitrogen Celleste 5.0 software (Thermo Fisher Scientific, Waltham, MA, USA). Four random regions of interest (ROIs) per kidney were analyzed per animal. Mean optical density (OD) was computed from the positively stained area relative to the total ROI area.

### 2.9. Renal CCL5 mRNA Quantification

To validate the transcriptomic findings, renal CCL5 mRNA expression was further analyzed using both conventional reverse transcription PCR (RT-PCR) and quantitative PCR (qPCR). Total RNA was extracted from renal tissues using the RNeasy Plus Universal Mini Kit (Qiagen, Hilden, Germany), and RNA purity was verified by spectrophotometry with A260/A280 ratios ranging from 1.9 to 2.1. cDNA was synthesized from 1 µg of total RNA using the iScript cDNA Synthesis Kit (Bio-Rad Laboratories, Inc., USA). For conventional RT-PCR, samples from five control and six MetS rats were used. Amplified CCL5 and internal control cyclophilin A (CycA) products were visualized on a 2% agarose gel stained with ethidium bromide. CycA was selected as the internal reference gene due to its stable expression in kidney tissue. All PCR experiments were performed within 24 h of RNA isolation to ensure transcript fidelity.

The qPCR analysis was conducted using the Applied Biosystems 7300 Real-Time PCR System (Foster City, CA, USA) with RT^2^ SYBR Green MasterMix (Qiagen). Relative mRNA expression was calculated using the 2^−ΔΔCt^ method, normalized to control values. The primer sequences for CCL5 were Forward 5′-GTG CCC ACG TGA AGG AGT AT-3′, Reverse 5′-GGA AAG CTA TAC AGG ATC A-3′; and for CycA, they were Forward 5′-TAT CTG CAC TGC CAA GAC TGA GTG-3′, Reverse 5′-CTT CTT GCT GGT CTT GCC ATT CC-3′.

### 2.10. Statistical Analysis

All continuous variables, including body weight, fasting glucose, serum lipid parameters, creatinine, renal blood flow, OxS markers (FORT/FORD), CCL5 mRNA, and protein levels, were expressed as mean ± standard error of the mean (SEM). The normality of data distribution was assessed using the Shapiro–Wilk test. For variables with normal distribution, comparisons between the MetS and control groups were performed using an unpaired two-tailed Student’s *t*-test. If a variable failed the normality test, appropriate non-parametric tests (e.g., Mann–Whitney U test) were applied. For PCR array-based differential gene expression analysis, individual gene *p*-values were further adjusted for multiple comparisons using the Holm–Bonferroni correction to control the family-wise error rate. Genes with Holm-adjusted *p*-values less than 0.05 were considered statistically significant. All analyses were conducted using GraphPad Prism 8.0 (GraphPad Software Inc., San Diego, CA, USA). A *p*-value less than 0.05 was considered statistically significant unless otherwise specified for multiple testing correction.

## 3. Results

### 3.1. Induction of MetS in Rats

#### 3.1.1. Weight Gain, Glucose Intolerance, and Insulin Resistance in MetS

**Figure 3:**
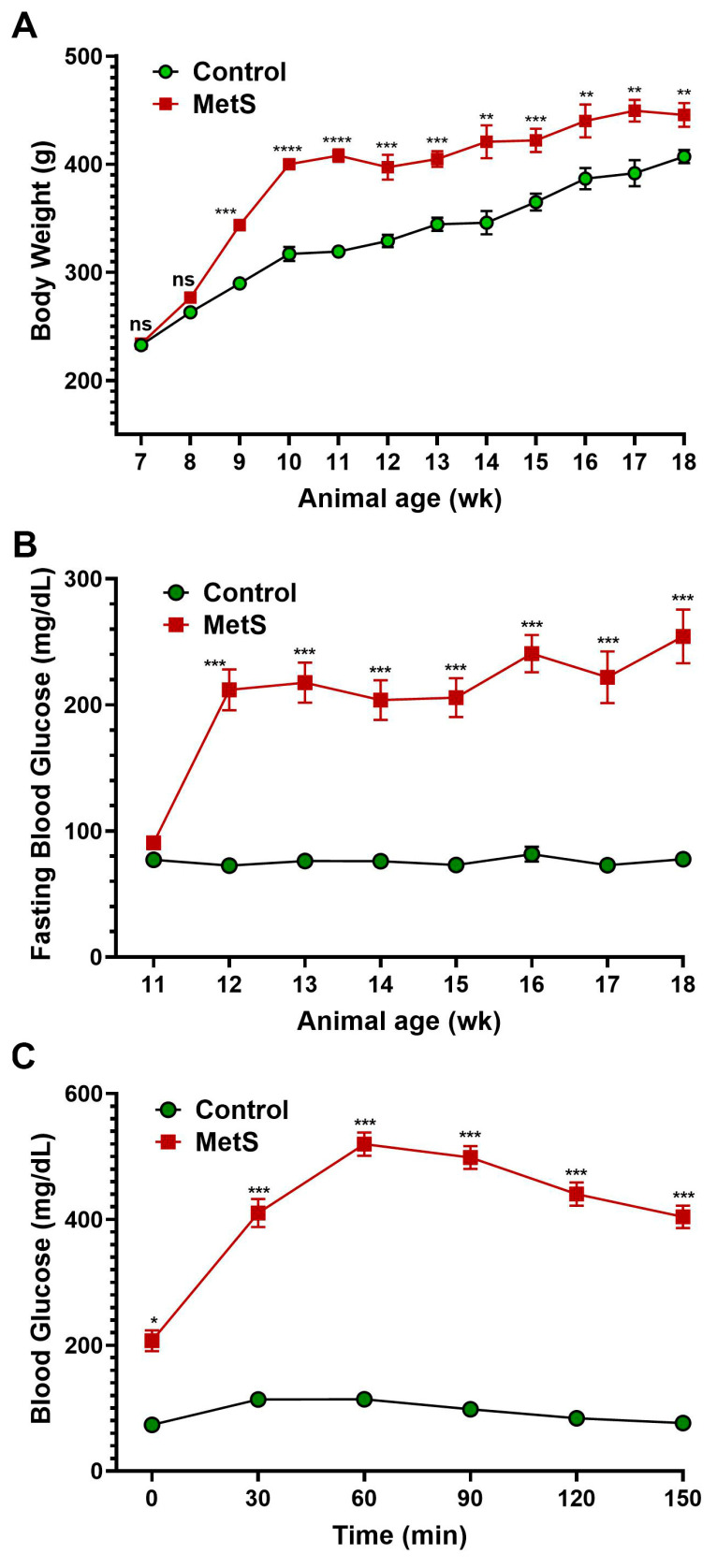
Changes in body weight and blood glucose in rats. (**A**) Body weight was recorded weekly throughout the experimental period to monitor obesity progression after metabolic syndrome (MetS) induction. (**B**) Fasting blood glucose levels were measured weekly from weeks 3 to 10 (animal age of wk 11 to 18) following an overnight fast to assess the development of hyperglycemia. (**C**) The oral glucose tolerance test (OGTT) was performed four weeks after streptozotocin injection. A glucose load (1.5 g/kg) was administered after a 12 h fast, and blood glucose levels were measured at 0, 30, 60, 90, 120, and 150 min to evaluate glucose tolerance. Control group: n = 20; MetS group: n = 30. * *p* < 0.05, ** *p* < 0.01, *** *p* < 0.001, and **** *p* < 0.0001 vs. control; ns = not significant.

The timeline summarized the experimental protocol for the establishment of MetS in rats (Figure 1). To evaluate the establishment of MetS, body weight and blood glucose parameters were monitored throughout the study (Figure 3). Rats fed an HFD showed markedly accelerated weight gain compared to controls, with significant differences emerging by week 9 and persisting until the end of the experiment (Figure 3A). After three weeks on the HFD, MetS rats received a single dose of STZ to induce insulin deficiency. Prior to the STZ injection (week 3), fasting blood glucose levels were comparable between groups and served as the baseline (Figure 3B). Following STZ administration, fasting glucose levels in the MetS group rose significantly and remained persistently elevated through week 10, indicating sustained hyperglycemia. In contrast, control rats maintained normoglycemia throughout. To assess glucose tolerance, an OGTT was conducted at week 7 (four weeks post-STZ injection). MetS rats demonstrated severely impaired glucose clearance, with blood glucose peaking at 60 min post-glucose load (519.9 ± 3.4 mg/dL) and remaining markedly elevated throughout the 150 min test period. Control rats, on the other hand, exhibited a normal glucose response with a rapid return to baseline (Figure 3C). These results confirm the successful induction of MetS, characterized by obesity, persistent hyperglycemia, and insulin resistance.

#### 3.1.2. Serum Lipid Profile

**Figure 4:**
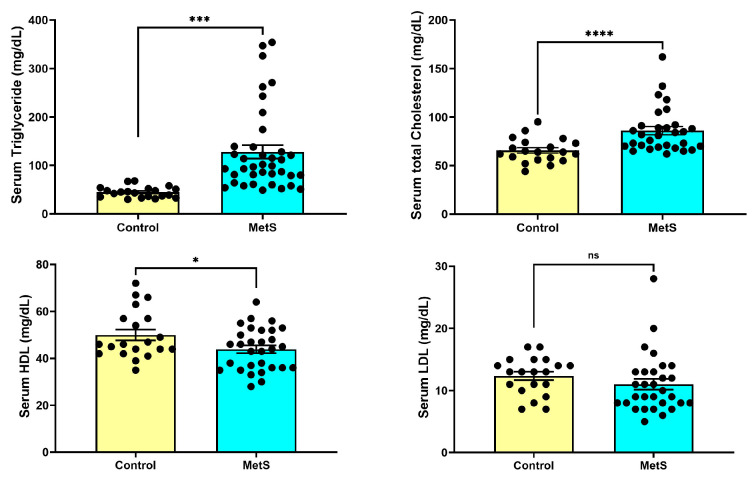
Changes in plasma lipid profile in rats. At week 7, plasma samples were collected to evaluate lipid metabolism alterations. Rats in the metabolic syndrome (MetS) group exhibited significantly elevated triglyceride (TG) levels compared to controls (**upper left panel**, *p* < 0.001). Total cholesterol (TC) levels were also markedly increased in the MetS group (**upper right panel**, *p* < 0.0001). In contrast, high-density lipoprotein (HDL) concentrations were modestly but significantly reduced (**lower left panel**, *p* < 0.05), while low-density lipoprotein (LDL) levels showed no significant difference between groups (**lower right panel**, *p* > 0.05). Data are presented as mean ± SEM in dot plots; control group, n = 20; MetS group, n = 30. * *p* < 0.05, *** *p* < 0.001, and **** *p* < 0.0001 vs. control; ns = not significant.

At week 7, rats in the MetS group exhibited significantly altered lipid parameters compared to controls (Figure 4). Serum TG levels were markedly elevated in MetS rats (128.0 ± 13.7 mg/dL) compared to the control group (44.80 ± 2.50 mg/dL, *p* < 0.001). TC concentrations were also significantly higher in MetS animals (86.03 ± 4.22 mg/dL) than in controls (65.65 ± 2.80 mg/dL, *p* < 0.0001). HDL levels were modestly but significantly decreased in the MetS group (43.90 ± 1.64 mg/dL vs. 50.00 ± 2.32 mg/dL in controls, *p* < 0.05). In contrast, LDL concentrations did not differ significantly between the two groups (*p* > 0.05). These metabolic alterations were accompanied by pronounced dyslipidemia, reflecting successful metabolic reprogramming induced by the HFD plus STZ.

#### 3.1.3. Changes in Hemodynamic and Body Parameters

**Figure 5:**
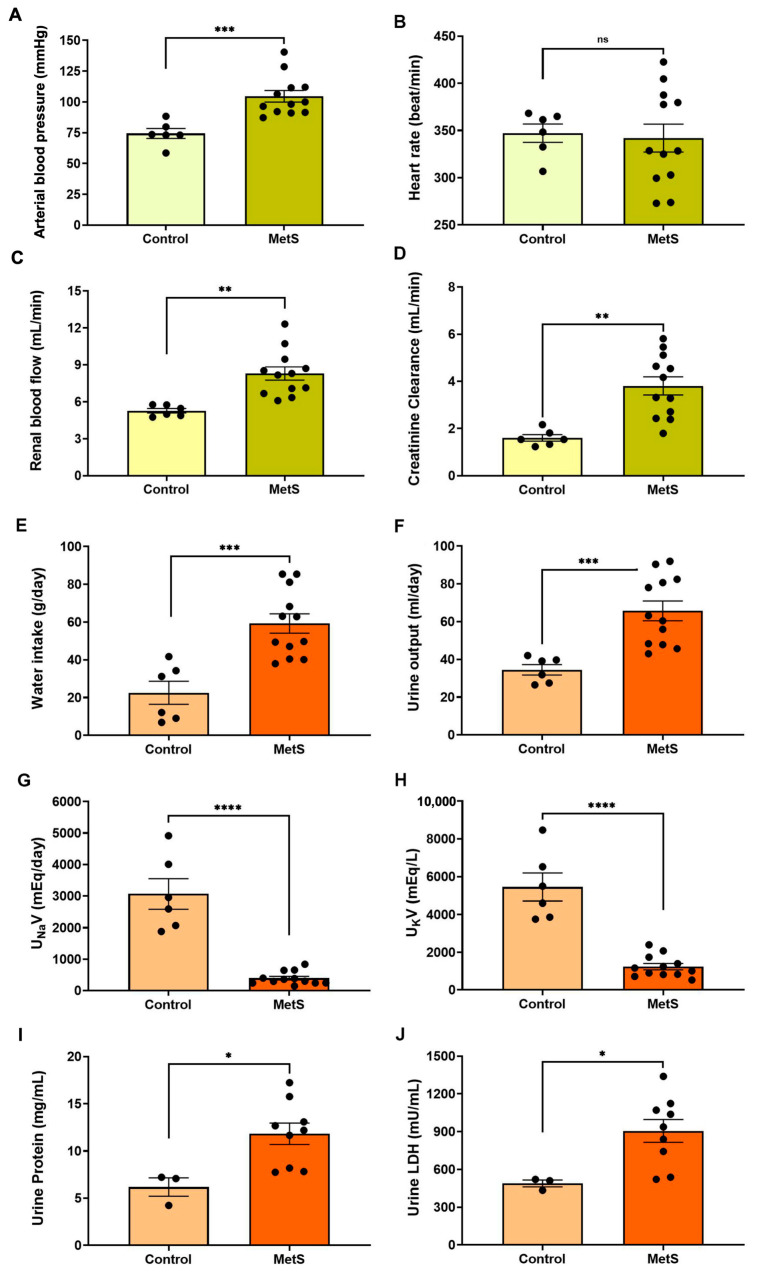
Cardiovascular and renal parameters in the metabolic syndrome (MetS) model. Hemodynamic and renal function parameters were evaluated in control and MetS rats prior to euthanasia. Arterial blood pressure (**A**), heart rate (**B**), renal blood flow (**C**), and creatinine clearance (CrCl) (**D**) were measured to evaluate systemic and renal hemodynamics. Water intake (**E**) and urine output (**F**) were recorded over a 24 h period. Urinary sodium excretion (U_Na_V, (**G**)) and urinary potassium excretion (U_K_V, (**H**)) were determined to assess changes in renal handling of electrolyte excretion in groups. Markers of renal injury included urine protein concentration (**I**) and urinary lactate dehydrogenase (LDH) activity (**J**). N = 6 (control) and n = 12 (MetS) for all parameters except urine protein and urinary LDH, for which n = 3 (control) and n = 9 (MetS). Data are presented as mean ± SEM. * *p* < 0.05, ** *p* < 0.01, *** *p* < 0.001, and **** *p* < 0.0001 vs. control; ns = not significant.

Hemodynamic evaluations conducted prior to euthanasia revealed a significant increase in mean arterial blood pressure in the MetS group compared to controls (104.6 ± 4.7 mmHg vs. 74.4 ± 4.0 mmHg, *p* < 0.001) (Figure 5A). Although the heart rate remained unchanged (Figure 5B), renal blood flow was markedly elevated in MetS rats (8.30 ± 0.54 mL/min vs. 5.27 ± 0.18 mL/min, *p* < 0.01) (Figure 5C), suggesting increased renal perfusion associated with early kidney dysfunction. CrCl was significantly increased (Figure 5D), consistent with glomerular hyperfiltration—a hallmark of early kidney injury. MetS rats also exhibited higher water intake (Figure 5E) and urine output (Figure 5F), along with reduced urinary sodium and potassium concentrations. In addition, urinary protein levels and LDH activity were significantly elevated in MetS rats, suggesting damage to tubular epithelial cells. Together, these findings indicate systemic fluid imbalance and renal dysfunction. Hemodynamic and renal assessments further support the successful establishment of a MetS model, recapitulating key features of metabolic and kidney pathology.

### 3.2. OxS-Related Renal Alterations in MetS Rats

#### 3.2.1. Systemic and Intrarenal OxS Is Elevated in MetS Rats

**Figure 6:**
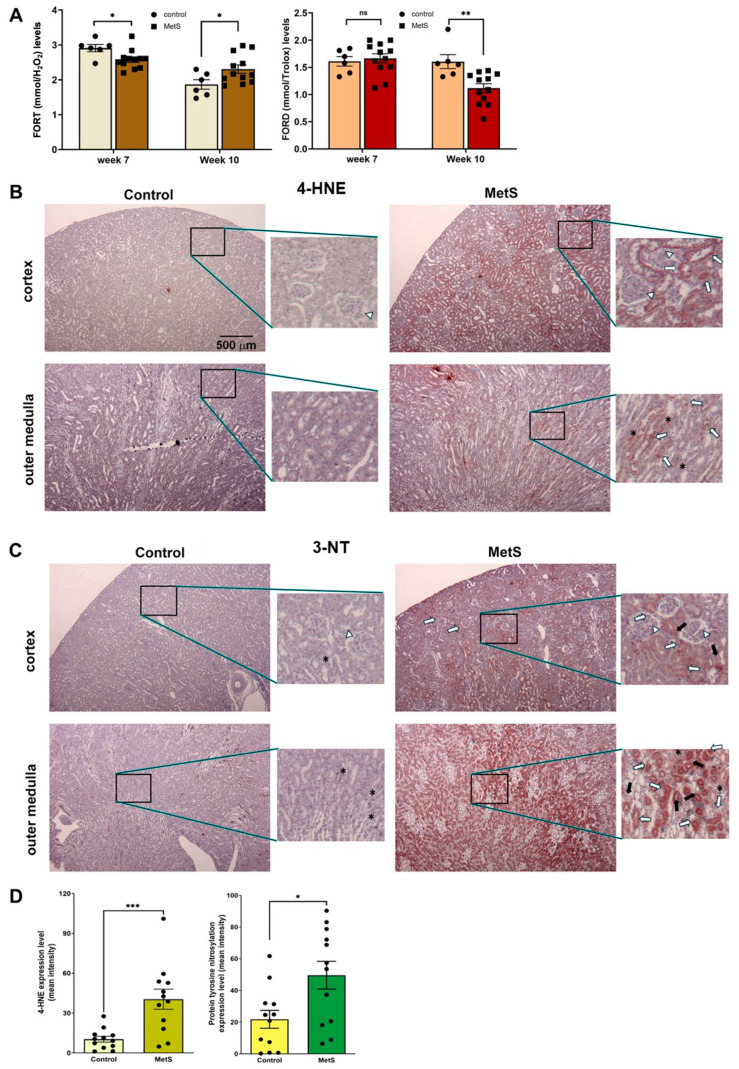
Oxidative stress status in serum and renal tissues. (**A**) Systemic oxidative stress was evaluated using the free oxygen radicals test (FORT) and the free oxygen radicals defense (FORD) assays at week 7, i.e., 4 weeks after STZ injection in the metabolic syndrome (MetS) group and week 10 (prior to sacrifice). Note that FORT levels at week 7 were slightly but significantly lower in MetS rats compared to controls, while FORD levels remained unchanged. By week 10, MetS rats exhibited significantly elevated FORT levels and reduced FORD levels relative to controls. Control group: n = 6; MetS group: n = 12. (**B**) Representative immunohistochemical staining of renal tissues at 40× magnification for a marker of lipid peroxidation, 4-hydroxynonenal (4-HNE), showed weak staining in the cortex and outer medulla of control rats, but it was markedly increased in MetS kidneys. (**C**) Representative immunohistochemical staining of renal tissues at 40× magnification for a marker of protein tyrosine nitration after OxS, 3-nitrotyrosine (3-NT), showed low levels in controls and strong expression in MetS kidneys, particularly in the tubules and interstitial regions. Insets in (**B**,**C**) show staining patterns at higher magnification (200×). The white arrows indicate glomeruli, long white arrows indicate proximal tubules, black arrows indicate distal tubules, and asterisks (*) denote interstitial cells. (**D**) Quantitative analysis of 4-HNE and 3-NT expression in renal tissue confirmed significantly higher OxS marker levels in MetS rats compared to controls. (n = 12 per group). Data are presented as mean ± SEM. * *p* < 0.05, ** *p* < 0.01, and *** *p* < 0.001 vs. control; ns = not significant.

To evaluate the OxS status in MetS, two standardized whole-blood assays were employed: the FORT and the FORD. The FORT provides a rapid assessment of circulating ROS, while the FORD measures overall antioxidant capacity by quantifying the reduction in preformed colored radicals, with absorbance inversely proportional to antioxidant levels [28]. At week 7 (midpoint of MetS induction), MetS rats exhibited a slight decrease in FORT levels and no significant change in FORD values compared to controls (Figure 6A). However, by week 10 (after 7 weeks of HFD + single-dose STZ treatment), FORT levels were significantly increased and FORD levels significantly decreased in MetS rats versus controls, indicating a progressive increase in systemic OxS.

Renal tissue analysis further revealed localized oxidative damage in MetS rats. Immunohistochemical staining for 4-HNE, a marker of lipid peroxidation, showed mild signal intensity in the renal cortex and outer medulla of control rats. In contrast, MetS rats demonstrated markedly stronger 4-HNE immunoreactivity in both regions, particularly in the outer medulla, where staining was most prominent (Figure 6B). The MetS-induced 4-HNE accumulation was most evident in the proximal and distal tubular segments (indicated by long white and black arrows, respectively), with an additional signal observed in glomeruli (white arrows) and interstitial cells (asterisks).

Similarly, immunostaining for 3-NT, a marker of protein tyrosine nitration and oxidative protein damage, showed low expression in control kidneys, with weak and diffuse staining in both the cortex and outer medulla. In MetS rats, however, 3-NT staining was clearly intensified, especially in the outer medulla, with widespread distribution in the proximal and distal tubules and interstitial compartments, as marked by the corresponding arrows in Figure 6C. Quantification revealed significantly higher 4-HNE and 3-NT levels in MetS rats compared to controls (Figure 6D).

#### 3.2.2. Downregulation of Redox-Related Genes in MetS Kidneys

To better characterize the oxidative transcriptomic alterations associated with MetS-induced renal injury, 84 OxS-related genes profiled in the PCR array were systematically categorized based on their subcellular localization, as listed in Table 1. A total of 57 genes encode cytoplasmic proteins, 5 genes encode molecules localized in the nucleus, 6 genes encode membrane-associated proteins, 5 genes encode proteins localized to mitochondria, 9 genes encode secreted proteins, and 2 genes encode extracellular proteins. Most of the redox molecules are present in multiple subcellular compartments, where they perform compartment-specific redox functions. Among them, six genes, including CCL5, glutamate-cysteine ligase catalytic subunit (Gclc), glutathione peroxidase 6 (Gpx6), recombination activating gene 2 (Rag2), NQO1, and SEPP1, were significantly suppressed, with fold changes greater than 2 (all *p* < 0.05). After Holm–Bonferroni correction for multiple comparisons, three of these genes—SEPP1 (adjusted *p* = 0.0002), CCL5 (adjusted *p* = 0.0043), and Rag2 (adjusted *p* = 0.0474)—remained statistically significant, highlighting their robust differential expression even under stringent control of false positives.

**Figure 7:**
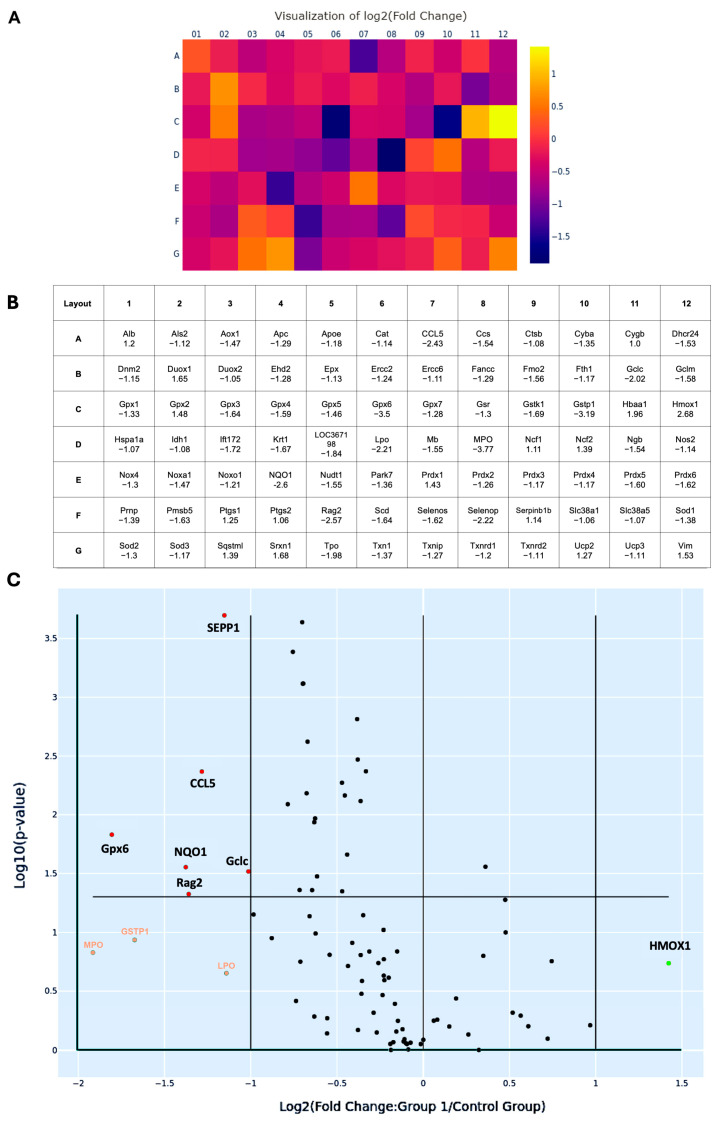
Changes in oxidative stress-related gene expression in renal tissues of rats with metabolic syndrome (MetS). (**A**) Heat map illustrating the log_2_ fold changes of 84 oxidative stress-associated genes in MetS versus control rats’ kidneys, based on quantitative PCR microarray analysis. Color scale represents gene expression levels: yellow indicates upregulation (positive fold change), while purple to dark blue indicates downregulation (negative fold change). (**B**) Corresponding gene layout table beneath the heat map, listing gene abbreviations (as in Table 1) and their respective fold change values. (**C**) Volcano plot displaying differentially expressed genes based on log_2_ fold change (*X*-axis) and –log_10_ (*p*-value in *Y*-axis). Genes with absolute fold change > 2 and *p* < 0.05 were considered significantly regulated. Statistically significant downregulated genes, including selenoprotein P-1 (SEPP1), C-C motif chemokine ligand 5 (CCL5), glutathione peroxidase 6 (Gpx6), glutamate-cysteine ligase catalytic subunit (Gclc), recombination activating gene 2 (Rag2), and NAD(P)H: quinone oxidoreductase 1 (NQO1), are shown in red dots. Genes with altered expression but not reaching statistical significance are heme oxygenase 1 (HMOX1), labeled as green, while glutathione S-transferase pi 1 (GSTP1), lactoperoxidase (LPO), and myeloperoxidase (MPO) are labeled orange. The horizontal line marks the *p*-value cutoff of 0.05. Control group: n = 6; MetS group: n = 12.

Heat map analysis revealed a predominant downregulation pattern of OxS-related genes in MetS kidneys (Figure 7A). In this visualization, each square represents an individual gene from the 84-target PCR array, arranged according to the physical 96-well layout (rows A–G, columns 01–12) (Figure 7B). Color gradients reflect log_2_ fold changes in expression (MetS vs. control), with purple to blue shades indicating downregulation and yellow to orange shades indicating upregulation. This layout allows rapid identification of expression trends across all tested genes in a spatially organized format. In the map, CCL5, Gclc, Gpx6, Rag2, NQO1, and SEPP1 show purple to dark blue colors with log_2_ fold changes > 1. These genes span key functional categories, including glutathione synthesis (as Gclc), mitochondrial detoxification (as Gpx6), redox cycling (as NQO1), selenium transport (as SEPP1), immune signaling (as CCL5), and DNA recombination (as Rag2), underscoring the multifaceted disruption of redox regulation in the MetS kidney. Similarly, volcano plot analysis provided a complementary visualization of statistical significance (–log_10_ *p*-value) versus magnitude of change (log_2_ fold change) across 84 genes (Figure 7C). This plot effectively distinguishes genes with both large expression shifts and strong statistical support, confirming that the majority of differentially expressed transcripts were downregulated under MetS conditions. Additionally, four genes exhibited more than twofold downregulation but this did not reach statistical significance. These included GSTP1, lactoperoxidase (LPO), myeloperoxidase (MPO), and heme oxygenase 1 (HMOX1). While not statistically significant, these trends may still reflect biologically relevant alterations in redox buffering under metabolic stress.

The multivariate analysis on supplementary data provides complementary evidence of a coordinated dysregulation of redox-related genes in the context of MetS. By applying partial least squares discriminant analysis (PLS-DA) to ΔCt-transformed gene expression data, we were able to distinguish MetS kidney samples from controls with high accuracy, highlighting the robustness of systemic redox disruption in this model (Figure S1). Notably, variable importance in projection (VIP) scores revealed that CCL5 ranked among the top contributors to group separation, reinforcing its potential biological significance in the pathogenesis of MetS-associated renal injury (Figure S2 and Table S1).

#### 3.2.3. Multi-Level Validation of CCL5 Suppression in Kidney and Circulation

**Figure 8:**
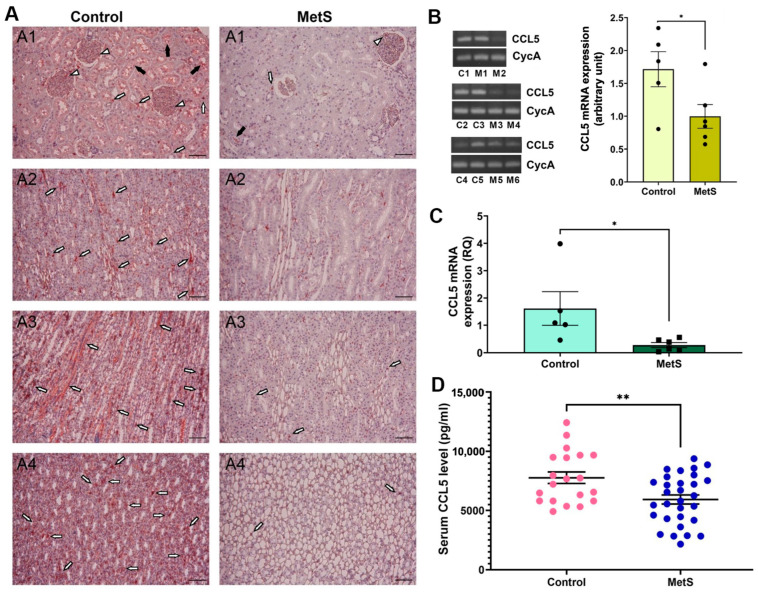
Multi-level validation of metabolic syndrome (MetS)-induced CCL5 downregulation in kidney and circulation. (**A**) Representative immunohistochemistry images revealed markedly lower CCL5 protein expression across renal compartments—including the cortex (**A1**), outer medulla (**A2**), inner medulla (**A3**), and papilla (**A4**)—in MetS rats compared to controls. White short arrows indicate glomerular CCL5 signals; white long arrows point to signals in the interstitial area between renal tubules; black arrows denote tubular luminal staining. Scale bar = 100 µm. (**B**) Conventional RT-PCR analysis of renal CCL5 mRNA expression. Left: representative gels show reduced CCL5 transcript levels (185 bp) in MetS kidneys compared to controls, with similar cyclophilin A (CycA, 127 bp) expression, used as an internal control. Each lane represents one animal: C1–C5 are control rats, and M1–M6 are MetS rats. Right: semi-quantitative analysis shows significantly decreased CCL5 expression in MetS rats relative to controls (control n = 5, MetS n = 6). (**C**) Quantitative PCR analysis of renal CCL5 mRNA expression. Relative quantification (RQ) demonstrates a significant reduction in CCL5 mRNA in the MetS group compared to controls (control n = 5, MetS n = 6). (**D**) Serum CCL5 concentrations measured by ELISA. Note that circulating CCL5 levels are significantly lower in MetS rats than in controls (control n = 20, MetS n = 30). All data are presented as mean ± SEM. * *p* < 0.05, and ** *p* < 0.01 vs. control.

We then sought to examine changes in CCL5 expression due to its known role in immune regulation and redox balance. CCL5 protein expression was examined in kidney tissues by immunohistochemistry (Figure 8A). In control rats, abundant CCL5 immunoreactivity was observed in glomerular areas (short white arrows), tubular lumina (black arrows), and interstitial compartments (long white arrows) across all major renal regions, including the cortex, outer medulla, inner medulla, and papilla. Notably, the outer medulla and papilla exhibited stronger staining intensity than the cortex, indicating region-specific enrichment under normal conditions. In contrast, MetS rats exhibited markedly diminished staining throughout the kidney, with the most pronounced signal loss in the outer medulla and papilla, indicating global suppression of renal CCL5 protein expression under metabolic stress. Next, conventional RT-PCR was used to assess renal CCL5 mRNA expression (Figure 8B, left). Representative gel bands targeting a 185 bp CCL5 fragment showed reduced intensity in MetS rats compared to controls, with similar CycA expression, used for normalization. Densitometric analysis confirmed a significant decrease in transcript abundance of CCL5 in MetS kidneys (Figure 8B, right). To further confirm this reduction, qPCR was performed on the same renal RNA samples, showing that CCL5 mRNA expression was significantly lower in MetS rats than in controls (Figure 8C). Finally, serum CCL5 concentrations were measured using ELISA to determine whether the intrarenal changes were also reflected systemically. Circulating CCL5 levels were significantly decreased in MetS rats (Figure 8D), supporting systemic suppression of CCL5 in the context of MetS.

## 4. Discussion

In this study, we successfully established a rat model of MetS by combining an HFD with a single-dose STZ injection. This model reproduced hallmark features of human MetS, including persistent hyperglycemia, glucose intolerance, dyslipidemia, obesity, elevated blood pressure, and renal hyperfiltration and injury. The progression of oxidative imbalance was confirmed by elevated serum FORT levels, reduced FORD capacity, and increased renal lipid and protein oxidation, as indicated by 4-HNE and 3-NT staining. Transcriptomic analysis of 84 OxS-related genes revealed broad transcriptional disturbances, with a dominant subset showing downregulation. Six genes—CCL5, Gclc, Gpx6, Rag2, NQO1, and SEPP1—were significantly suppressed. CCL5 downregulation was further validated at the mRNA, protein, and serum levels using RT-PCR, qPCR, immunohistochemistry, and ELISA, supporting its potential as a robust biomarker for MetS-induced renal damage.

The combination of HFD and STZ induces insulin resistance and pancreatic β-cell dysfunction, mimicking the dual pathophysiological mechanisms of type 2 diabetes. STZ impairs insulin secretion [29], while the HFD impairs insulin signaling and promotes triglyceride accumulation [30,31]. Our data demonstrated that this combined regimen resulted in significant hyperglycemia, hypertriglyceridemia, and increased total cholesterol without corresponding LDL elevation (Figure 3 and Figure 4). These findings, together with previous reports, suggest enhanced hepatic VLDL production—a characteristic feature of insulin resistance and MetS [32]. Wistar rats were chosen in this study for their higher susceptibility to HFD-induced obesity than Sprague-Dawley rats, attributed to elevated cluster of differentiation 36 (CD36) and lipoprotein lipase expression [33]. Collectively, this dual-intervention approach effectively recapitulates the hallmark features of human MetS, including metabolic dysregulation and associated target organ damage. Consistent with prior studies, our findings confirm that the HFD/STZ model is a reliable and translationally relevant tool for investigating MetS-induced renal complications.

In our model, significantly increased CrCl indicated glomerular hyperfiltration, while elevated urinary LDH and proteinuria suggested tubular and glomerular injury (Figure 5). Not only hemodynamic deterioration, but also impaired renal excretion of salt may contribute to the development or maintenance of hypertension, further exacerbating a systemic and intrarenal pressure load for target organ damage [34]. These maladaptive changes can promote fluid retention, increase blood volume, and perpetuate vascular injury, ultimately leading to target organ damage, including the kidneys [35,36]. These findings support the notion that MetS accelerates renal deterioration, with urinary protein excretion serving as a sensitive and quantifiable marker of injury progression. In addition, increased renal blood flow and elevated blood pressure were also observed, both of which have been implicated in target organ damage in previous hypertensive or MetS-related models [37,38,39]. Sustained renal hyperperfusion and hypertension can exacerbate glomerular pressure and shear stress, ultimately contributing to glomerulosclerosis and tubular injury. Together with OxS, these hemodynamic disturbances likely act synergistically to drive progressive renal dysfunction in the context of MetS.

FORT and FORD assays have been successfully applied in several clinical studies, including in acute myocardial infarction, type 2 diabetes, sickle cell disease, and obesity, supporting their relevance for assessing systemic OxS in cardiometabolic conditions [28,40,41,42]. These findings indicate that the FORT and FORD can sensitively capture redox imbalances in chronic metabolic states. Compared to classical methods such as TBARS (thiobarbituric acid reactive substances) and FRAP (ferric reducing antioxidant power), the FORT and FORD offer distinct analytical advantages. The TBARS assay, which measures late-stage lipid peroxidation products such as malondialdehyde (MDA), suffers from low specificity due to interference by sugars and amino-containing compounds [43]. In contrast, the FORT detects hydroperoxides as early-phase oxidative markers, providing higher specificity and earlier detection of redox imbalance [28]. Likewise, FRAP, while widely used to assess total reducing capacity under acidic conditions, does not reflect radical scavenging activity and fails to detect thiol-based antioxidants [44,45,46]. In comparison, the FORD measures non-enzymatic antioxidant defense at a near-physiological pH, better reflecting the dynamic antioxidant reserve during OxS [28,42]. These methodological distinctions reinforce the rationale for using the FORT and FORD in this study, particularly in a MetS model characterized by progressive oxidative dysfunction and metabolic overload.

The resultant metabolic disturbances synergistically intensified OxS (Figure 6). Nutrient overload induced mitochondrial ROS generation, while adipocyte hypertrophy and chronic inflammation amplified the systemic oxidative burden [47,48]. Hyperglycemia further elevated ROS through increased polyol flux, protein glycation, and mitochondrial impairment, while dyslipidemia contributed to lipid peroxidation and the accumulation of oxidized lipoproteins [49,50]. These mechanisms collectively overwhelmed antioxidant defenses, disrupted redox homeostasis, and fostered renal oxidative injury. Renal OxS plays a central role in the pathogenesis of kidney disease, driving glomerular and tubular injury and enhancing proteinuria [51]. Across species, mitochondria, NADPH oxidase (NOX), and inducible nitric oxide synthase (iNOS) are primary sources of ROS and RNS, mediating both acute and chronic kidney injury [52]. Increased systemic and renal OxS in the MetS model was evidenced by significantly elevated serum FORT levels and enhanced renal expression of lipid and protein oxidation markers, including 4-HNE and 3-NT. Notably, temporal analysis revealed that at week 7, FORT levels were mildly reduced and FORD remained unchanged, whereas by week 10, FORT levels had significantly increased and FORD levels declined. These changes indicate a systemic transition toward uncompensated OxS and impaired redox-buffering capacity. These results align with previous studies in MetS models, which reported that redox imbalance begins as a mild or compensatory response but progressively worsens as the disease advances [53,54], with transient activation of antioxidant defenses in early-stage HFD-induced metabolic stress often followed by their depletion and subsequent oxidative damage in later phases.

Further supporting this shift, transcriptomic profiling revealed that six OxS-related genes were significantly downregulated, with none being significantly upregulated in MetS kidneys (Table 1 and Figure 7). Interestingly, the redox proteins encoded by these genes have different subcellular locations and functions. For example, CCL5, a secreted chemokine, exerts antioxidant effects in the extracellular environment by activating heme oxygenase-1, thereby attenuating ROS production [55]. Gclc, located in the cytoplasm and mitochondria, is the rate-limiting enzyme in glutathione biosynthesis and generates intracellular glutathione, a major cellular antioxidant that defends against oxidative insults [56]. Gclc is also positively regulated by insulin via the Nrf2 pathway, enhancing antioxidant capacity [57]. NQO1, an NAD(P)H-dependent flavoprotein, detoxifies quinones and prevents redox cycling, thereby suppressing ROS generation [58,59]. Its overexpression has been shown to enhance NAD⁺/NADH ratios, activate Sirt1, and promote Forkhead box O (FOXO)- and peroxisome proliferator-activated receptor gamma coactivator 1-α (PGC-1α)-mediated antioxidant defenses [60]. NQO1, also found in the cytoplasm, catalyzes two-electron reductions of quinones to hydroquinones, thereby preventing redox cycling and limiting ROS generation; it also contributes to the regeneration of ubiquinol and α-tocopherol hydroquinone, reinforcing intracellular antioxidant capacity [59]. Gpx6, along with other glutathione peroxidases (Gpx1–4), detoxifies peroxides and maintains redox balance [13]. Rag2, typically known for its role in lymphocyte development, also exhibits peroxidase-like activity under OxS, particularly in ischemic models, suggesting its protective role [61]. SEPP1, a secreted glycoprotein, provides systemic antioxidant protection by transporting selenium and scavenging extracellular ROS. Additionally, SEPP1 safeguards LDL from peroxidative damage, reduces peroxynitrite, and facilitates the intracellular synthesis of other antioxidant selenoproteins via receptor-mediated uptake through ApoER2 and megalin [62]. Deficiency in SEPP1 impairs immune and epithelial cell function, predisposing tissues to inflammatory tumorigenesis [63].

The co-occurrence of CCL5 with canonical antioxidant genes such as Gpx6, NQO1, and Gclc, which are among the top-ranked VIP contributors, suggests that the transcriptomic response to MetS encompasses not only classical oxidative enzymes but also immunomodulatory and redox-regulatory mediators. Given the previously established role of CCL5 as an inflammation-related chemokine, these findings support a systems-level interpretation in which OxS and inflammation may synergistically compromise redox homeostasis. Although the specific mechanisms by which CCL5 modulates these pathways remain to be elucidated, our use of a multivariate analytical framework offers a more integrated view of the underlying gene interactions and underscores the utility of network-informed approaches in identifying functionally interconnected biomarkers.

Among the downregulated genes, CCL5 stands out due to its dual roles in inflammation and tissue protection. While CCL5 is widely recognized for promoting immune cell migration and has been implicated in chronic diseases, cancers, and viral infections such as COVID-19 [64,65,66], it also possesses antioxidant and tissue-repair functions under specific pathological conditions [24,67,68]. Notably, CCL5 deficiency has been shown to protect against adipose tissue inflammation in obesity [69,70], yet it has also been shown to exacerbate kidney injury in hypertensive models [25]. Moreover, the deletion of its receptor, CCR5, alters macrophage polarization and improves insulin sensitivity [71]. In contrast, CCL5 deficiency itself has been associated with aggravated adipose inflammation and metabolic impairment [22]. Similar CCL5 downregulation has also been observed in patients with Alzheimer’s disease [72]. These context-dependent roles support the “double-edged sword” hypothesis of the CCR5-CCL5 axis in MetS and cardiovascular disease [70]. In our findings, the consistent downregulation of CCL5 across RT-PCR, qPCR, and ELISA strongly supports its potential as a robust biomarker for MetS-associated renal damage (Figure 8). Although the underlying mechanisms remain unclear, OxS-mediated inhibition of chemokine signaling represents a plausible explanation warranting further investigation. Supporting this, dynamic changes in systemic OxS were observed in the MetS model.

Interestingly, several other OxS-related genes previously reported to be significantly altered in MetS were either unchanged or downregulated in our model, emphasizing the complexity and tissue-specific nature of renal oxidative responses. Although NOX isoforms such as NOX4 are widely recognized as major sources of ROS in the kidney under metabolic stress [52], our transcriptomic analysis did not reveal significant upregulation of NOX4 (Table 1 and Figure 7). Likewise, similar findings to this study were reported in a HFD-induced MetS model, where NOX4 deletion did not provide metabolic protection, nor was NOX4 expression markedly elevated in major metabolic organs such as the kidney, liver, or heart [73]. These results suggest that under chronic metabolic stress, ROS production may shift toward alternative sources—such as mitochondrial dysfunction or xanthine oxidase—or that NOX4-derived hydrogen peroxide serves a regulatory rather than a damaging role. Moreover, compensatory feedback loops or adaptive transcriptional control may limit NOX4 expression as a protective mechanism against excessive oxidative injury. This aligns with the broader pattern observed in our study, where several canonical oxidative genes failed to exhibit the anticipated upregulation, highlighting the dynamic and context-dependent regulation of redox pathways in MetS-associated renal injury.

In line with OxS, catalase, a key antioxidant enzyme frequently reported to be reduced in MetS patients, remained stable in our model. Similar findings were also observed in HFD-induced MetS mouse models, where hepatic catalase activity was preserved despite elevated lipid peroxidation and metabolic dysfunction [74]. This apparent stability may reflect organ-specific redox buffering or an adaptive threshold beyond which catalase expression is no longer inducible under chronic OxS. Likewise, MPO, typically elevated in obesity-related inflammatory and oxidative states, exhibited only a non-significant downward trend in our model. This subtle or absent elevation may indicate limited leukocyte recruitment or renal-specific immune modulation. Supporting this, a clinical cross-sectional study reported significantly higher MPO levels only in individuals with nondiabetic MetS, but not in those with prediabetes or newly diagnosed type 2 diabetes [75]. These observations suggest that MPO activation may peak during early metabolic derangements and wane as hyperglycemia progresses, potentially explaining the modest MPO expression observed in our model. Together, these findings underscore the importance of temporal dynamics and organ-specific regulation in shaping OxS responses during MetS progression.

In summary, our data reveal that systemic and renal OxS, along with coordinated downregulation of antioxidant and redox-regulatory genes, particularly CCL5, contribute significantly to MetS-associated kidney injury. The absence of expected upregulation in canonical OxS genes further underscores the complexity and temporal variability of redox responses in this condition. Future studies incorporating longitudinal sampling and organ-specific profiling are warranted to better characterize the redox landscape of metabolic kidney disease and to identify actionable therapeutic targets or biomarkers.

Nonetheless, the study has certain limitations. All animals used were male Wistar rats, and potential sex-based differences in metabolic and oxidative responses were not addressed. The follow-up duration was limited to 10 weeks post-induction, which may not fully capture the chronic progression of renal pathology in MetS. Additionally, our analyses focused primarily on the kidney, while systemic oxidative changes and involvement of other target organs—such as the liver or heart—were not explored. Future studies should consider including both sexes, extending the observation period, and incorporating multi-organ evaluations to better elucidate the systemic impact of redox dysregulation in MetS.

## 5. Conclusions

In this study, we successfully established a MetS rat model using an HFD combined with a single STZ injection, reproducing key clinical features such as obesity, hyperglycemia, insulin resistance, dyslipidemia, and renal injury. OxS emerged as a major contributor to kidney damage, as evidenced by elevated lipid peroxidation and protein oxidation. Transcriptomic profiling revealed significant downregulation of six antioxidant genes—CCL5, Gclc, Gpx6, Rag2, NQO1, and SEPP1—highlighting their potential contribution to redox imbalance. Among them, CCL5, a chemokine implicated in immune regulation and tissue repair, was consistently suppressed at both mRNA and protein levels in renal tissues and serum. These findings suggest that CCL5 may serve a protective role in maintaining renal redox homeostasis, and its reduction may exacerbate oxidative injury under MetS conditions.

## Figures and Tables

**Table 1:** The expression profile for the 84 oxidative stress genes.

Encoded Molecules in Subcellular Location	Gene	Fold Regulation	*p*-Value
Cytoplasm (for 57 genes)	Amyotrophic lateral sclerosis 2 (juvenile) homolog (Als2) ^%^	−1.12	0.4056
	Aldehyde oxidase 1 (Aox1)	−1.47	0.7197
	Adenomatous polyposis coli (Apc) ^#^	−1.29	0.0076 **
	Catalase (Cat)	−1.14	0.8825
	Copper chaperone for superoxide dismutase (Ccs)	−1.54	0.0107 *
	Cathepsin B (Ctsb) ^#$&^	−1.08	0.8053
	Cytoglobin (Cygb) ^%^	1.00	0.8149
	24-dehydrocholesterol reductase (Dhcr24)	−1.53	0.0335 *
	Dynamin 2 (Dnm2) ^#^	−1.15	0.2431
	EH-domain containing 2 (Edh2) ^#^	−1.28	0.2593
	Fanconi anemia, complementation group C (Fancc) ^%^	−1.29	0.1559
	Flavin containing monooxygenase 2 (Fmo2)	−1.56	0.0439 *
	Ferritin, heavy polypeptide 1 (Fth1)	−1.17	0.1693
	Glutamate-cysteine ligase, catalytic subunit (Gclc) ^@^	**−2.02**	**0.0304** *
	Glutamate cysteine ligase, modifier subunit (Gclm)	−1.58	0.0731
	Glutathione peroxidase 1 (Gpx1) ^@^	−1.33	0.1227
	Glutathione peroxidase 2 (Gpx2)	1.48	0.5107
	Glutathione peroxidase 4 (Gpx4) ^%@^	−1.59	0.0024 **
	Glutathione peroxidase 7 (Gpx7)	−1.28	0.3334
	Glutathione reductase (Gsr) ^@^	−1.30	0.0034 **
	Glutathione S-transferase pi 1 (Gstp1) ^%@^	−3.19	0.1159
	Heme oxygenase (decycling) 1 (Hmox1; HO-1)	2.68	0.1832
	Heat shock 70kD protein 1A (Hspa1a) ^%$^	−1.07	0.8719
	Isocitrate dehydrogenase 1 (NADP+), soluble (Idh1)	−1.08	0.8409
	Keratin 1 (Krt1) ^#^	−1.67	0.3836
	Myoglobin (Mb)	−1.55	0.5192
	Neutrophil cytosolic factor 1 (Ncf1) ^#^	1.11	0.6298
	Neutrophil cytosolic factor 2 (Ncf2)	1.39	0.1001
	Neuroglobin (Ngb) ^@^	−1.54	0.1022
	Nitric oxide synthase 2, inducible (Nos2)	−1.14	0.9919
	NADPH oxidase 4 (NOX4) ^#^	−1.30	0.6739
	NADPH oxidase activator 1 (Noxa1) ^#^	−1.47	0.5365
	NADPH oxidase organizer 1 (Noxo1)	−1.21	0.7064
	NAD(P)H dehydrogenase, quinone 1 (NQO1)	**−2.60**	**0.0279** *
	Nudix (nucleoside diphosphate linked moiety X)-type motif 1 (Nudt1)	−1.55	0.0116 *
	Parkinson’s disease (autosomal recessive, early onset) 7 (Park7) ^@#^	−1.36	0.0218 *
	Peroxiredoxin 1 (Prdx1)	1.43	0.4819
	Peroxiredoxin 2 (Prdx2)	−1.26	0.0043 **
	Peroxiredoxin 3 (Prdx3) ^@^	−1.17	0.2556
	Peroxiredoxin 4 (Prdx4) ^$^	−1.17	0.0955
	Peroxiredoxin 5 (Prdx5) ^@^	−1.60	0.0066 **
	Peroxiredoxin 6 (Prdx6)	−1.62	0.0008 ***
	Prion protein (Prnp)	−1.39	0.0053 **
	Proteasome (prosome, macropain) subunit, beta type 5 (Psmb5) ^%^	−1.63	0.0002 ***
	Prostaglandin-endoperoxide synthase 1 (Ptgs1)	1.25	0.9918
	Prostaglandin-endoperoxide synthase 2 (Ptgs2) ^%^	1.06	0.5522
	Stearoyl-CoA desaturase (Scd) ^#^	−1.64	0.1776
	Selenoprotein S (Selenos)	−1.62	0.0008 ***
	Serine (or cysteine) peptidase inhibitor, clade B, member 1b (Serpinb1b)	1.14	0.3653
	Superoxide dismutase 1, soluble (SOD1) ^%^	−1.38	0.0449 *
	Superoxide dismutase 3, extracellular (SOD3) ^$^	−1.17	0.2335
	Sequestosome 1 (Sqstm1) ^%^	1.39	0.0530
	Sulfiredoxin 1 homolog (S. cerevisiae) (Srxn1)	1.68	0.1758
	Thioredoxin 1 (Txn1) ^%$^	−1.37	0.0069 **
	Thioredoxin interacting protein (Txnipp)	−1.27	0.0717
	Thioredoxin reductase 1 (Txnrd1)	−1.20	0.1823
	Vimentin (Vim) ^%^	1.53	0.6269
Nucleus (for 5 genes)	Excision repair cross-complementing rodent repair deficiency, complementation group 2 (Ercc2)	−1.24	0.1454
	Excision repair cross-complementing rodent repair deficiency, complementation group 6 (Ercc6)	−1.11	0.5644
	Intraflagellar transport 172 homolog (Chlamydomonas) (Ift172)	−1.72	0.0081 **
	Similar to Serine/threonine-protein kinase ATR (Ataxia telangiectasia and Rad3-related protein) (LOC367198)	−1.84	0.1119
	Recombination activating gene 2 (Rag2)	**−2.57**	**0.0474** *
Membrane-associated (for 6 genes)	Dual oxidase 1 (Duox1)	1.65	0.7964
	Dual oxidase 2 (Duox2)	−1.05	0.8609
	Glutathione S-transferase kappa 1 (Gstk1)	−1.69	0.0004 ***
	Solute carrier family 38, member 1 (Slc38a1)	−1.06	0.9819
	Solute carrier family 38, member 5 (Slc38a5)	−1.07	0.8834
	Thyroid peroxidase (Tpo)	−1.98	0.0707
Mitochondria (for 5 genes)	Cytochrome b-245, alpha polypeptide (Cyba)	−1.35	0.1932
	Uncoupling protein 2 (mitochondrial, proton carrier) (Ucp2)	1.27	0.1585
	Uncoupling protein 3 (mitochondrial, proton carrier) (Ucp3)	−1.11	0.1453
	Superoxide dismutase 2, mitochondrial (SOD2)	−1.30	0.0015 **
	Thioredoxin reductase 2 (Txnrd2)	−1.11	0.6944
Secreted (for 9 genes)	Albumin (Alb)	1.20	0.7347
	Apolipoprotein E (Apoe) ^&^	−1.18	0.3419
	Chemokine (C-C motif) ligand 5 (CCl5)	**−2.43**	**0.0043** **
	Glutathione peroxidase 3 (Gpx3)	−1.64	0.0438 *
	Glutathione peroxidase 5 (Gpx5)	−1.46	0.1551
	Glutathione peroxidase 6 (Gpx6)	**−3.50**	**0.0148** *
	Lactoperoxidase (LPO)	−2.21	0.2226
	Myeloperoxidase (MPO) ^&^	−3.77	0.1487
	Selenoprotein P-1 (SEPP1)	**−2.22**	**0.0002** ***
Extracellular space (for 2 genes)	Eosinophil peroxidase (Epx)	−1.13	0.8530
	Hemoglobin alpha, adult chain 1 (Hba-a1)	1.96	0.6148

Note: Values in bold red indicate genes with fold change > 2 and *p <* 0.05. Several genes have multiple localizations, as indicated by superscript symbols: ^%^ also distributed in the nucleus; ^@^ also distributed in the mitochondria; ^#^ also distributed in cell membrane; ^$^ can be acting as a secretory protein; ^&^ also distributed in extracellular space; * *p* < 0.05, ** *p* < 0.01, and *** *p* < 0.001 vs. control.

## Data Availability

The data presented in this study are available on request from the corresponding author due to institutional data protection and ethical considerations related to animal research.

## Supplementary Materials

The following supporting information is available at https://www.mdpi.com/article/10.3390/antiox14060746/s1. Figure S1: Partial least squares discriminant analysis (PLS-DA). Figure S2. Variable importance in projection (VIP) score plot. Table S1. List of VIP scores from PLS-DA model for six downregulated genes.

## Abbreviations

3-NT	3-Nitrotyrosine
4-HNE	4-Hydroxynonenal
CAT	Catalase
CCR	CC chemokine receptor
CCL5	C-C motif chemokine ligand 5
CKD	Chronic kidney disease
CrCl	Creatinine clearance
CycA	Cyclophilin A
DEG	Differentially expressed gene
ELISA	Enzyme-linked immunosorbent assay
FBG	Fasting blood glucose
FBS	Fetal bovine serum
FOXO	Forkhead box O
FORT	Free oxygen radicals test
FORD	Free oxygen radicals defense
GSTP1	Glutathione S-transferase pi 1
GAPDH	Glyceraldehyde-3-phosphate dehydrogenase
Gpx6	Glutathione peroxidase 6
Gclc	Glutamate-cysteine ligase catalytic subunit
HDL	High-density lipoprotein
HFD	High-fat diet
HMOX1	Heme oxygenase 1
iNOS	Inducible nitric oxide synthase
LDH	Lactate dehydrogenase
LDL	Low-density lipoprotein
LPO	Lactoperoxidase
MetS	Metabolic syndrome
MPO	Myeloperoxidase
NOX	NADPH oxidases
NQO1	NAD(P)H: quinone oxidoreductase 1
OGTT	Oral glucose tolerance test
OxS	Oxidative stress
PBS	Phosphate-buffered saline
PGC-1α	Peroxisome proliferator-activated receptor gamma coactivator 1-alpha
qPCR	Quantitative PCR
Rag2	Recombination activating gene 2
RANTES	Regulated on activation, normal T cell expressed and secreted (alias of CCL5)
RNS	Reactive nitrogen species
ROS	Reactive oxygen species
RT-PCR	Reverse transcription PCR
ROI	Region of interest
SEPP1	Selenoprotein P-1
SEM	Standard error of the mean
SOD2	Superoxide dismutase 2
STZ	Streptozotocin
TC	Total cholesterol
TG	Triglyceride
TGF-β1	Transforming growth factor-β1
VLDL	Very-low-density lipoprotein

## References

[1] James P.T., Rigby N., Leach R. (2004). The obesity epidemic, metabolic syndrome and future prevention strategies. Eur. J. Cardiovasc. Prev. Rehabil..

[2] Locatelli F., Pozzoni P., Del Vecchio L. (2006). Renal manifestations in the metabolic syndrome. J. Am. Soc. Nephrol..

[3] Spahis S., Borys J.-M., Levy E. (2017). Metabolic Syndrome as a Multifaceted Risk Factor for Oxidative Stress. Antioxid. Redox Signal..

[4] Yang L., Chang B., Guo Y., Wu X., Liu L. (2019). The role of oxidative stress-mediated apoptosis in the pathogenesis of uric acid nephropathy. Ren. Fail..

[5] Chen J., Zhang H., Yi X., Dou Q., Yang X., He Y., Chen J., Chen K. (2024). Cellular senescence of renal tubular epithelial cells in acute kidney injury. Cell Death Discov..

[6] Fakhruddin S., Alanazi W., Jackson K.E. (2017). Diabetes-Induced Reactive Oxygen Species: Mechanism of Their Generation and Role in Renal Injury. J. Diabetes Res..

[7] Irazabal M.V., Torres V.E. (2020). Reactive Oxygen Species and Redox Signaling in Chronic Kidney Disease. Cells.

[8] Rapa S.F., Di Iorio B.R., Campiglia P., Heidland A., Marzocco S. (2019). Inflammation and Oxidative Stress in Chronic Kidney Disease-Potential Therapeutic Role of Minerals, Vitamins and Plant-Derived Metabolites. Int. J. Mol. Sci..

[9] Zhang X., Lerman L.O. (2017). The metabolic syndrome and chronic kidney disease. Transl. Res..

[10] Prasad G.V. (2014). Metabolic syndrome and chronic kidney disease: Current status and future directions. World. J. Nephrol..

[11] Tesauro M., Canale M.P., Rodia G., Di Daniele N., Lauro D., Scuteri A., Cardillo C. (2011). Metabolic syndrome, chronic kidney, and cardiovascular diseases: Role of adipokines. Cardiol. Res. Pract..

[12] Zhang H., Forman H.J. (2012). Glutathione synthesis and its role in redox signaling. Semin. Cell Dev. Biol..

[13] Pei J., Pan X., Wei G., Hua Y. (2023). Research progress of glutathione peroxidase family (GPX) in redoxidation. Front. Pharmacol..

[14] Rashid M.H., Babu D., Siraki A.G. (2021). Interactions of the antioxidant enzymes NAD(P)H: Quinone oxidoreductase 1 (NQO1) and NRH: Quinone oxidoreductase 2 (NQO2) with pharmacological agents, endogenous biochemicals and environmental contaminants. Chem. Biol. Interact..

[15] Steinbrenner H., Sies H. (2009). Protection against reactive oxygen species by selenoproteins. Biochim. Biophys. Acta..

[16] He L., He T., Farrar S., Ji L., Liu T., Ma X. (2017). Antioxidants Maintain Cellular Redox Homeostasis by Elimination of Reactive Oxygen Species. Cell. Physiol. Biochem..

[17] Shang Y., Siow Y.L., Isaak C.K., O K. (2016). Downregulation of Glutathione Biosynthesis Contributes to Oxidative Stress and Liver Dysfunction in Acute Kidney Injury. Oxidative Med. Cell. Longev..

[18] Nadkarni G.D., Sawant B.U. (1998). Role of antioxidant defence in renal pathophysiology: Primacy of glutathione peroxidase. Indian J. Exp. Biol..

[19] Gang G.T., Kim Y.H., Noh J.R., Kim K.S., Jung J.Y., Shong M., Hwang J.H., Lee C.H. (2013). Protective role of NAD(P)H:quinone oxidoreductase 1 (NQO1) in cisplatin-induced nephrotoxicity. Toxicol. Lett..

[20] Li S., Zhao Q., Zhang K., Sun W., Jia X., Yang Y., Yin J., Tang C., Zhang J. (2020). Se deficiency induces renal pathological changes by regulating selenoprotein expression, disrupting redox balance, and activating inflammation. Metallomics.

[21] Mir M.M., Alfaifi J., Sohail S.K., Rizvi S.F., Akhtar M.T., Alghamdi M.A.A., Mir R., Wani J.I., Sabah Z.U., Alhumaydhi F.A. (2024). The Role of Pro-Inflammatory Chemokines CCL-1, 2, 4, and 5 in the Etiopathogenesis of Type 2 Diabetes Mellitus in Subjects from the Asir Region of Saudi Arabia: Correlation with Different Degrees of Obesity. J. Pers. Med..

[22] Zhou H., Liao X., Zeng Q., Zhang H., Song J., Hu W., Sun X., Ding Y., Wang D., Xiao Y. (2022). Metabolic effects of CCL5 deficiency in lean and obese mice. Front. Immunol..

[23] Liu X., Shah A., Gangwani M.R., Silverstein P.S., Fu M., Kumar A. (2014). HIV-1 Nef induces CCL5 production in astrocytes through p38-MAPK and PI3K/Akt pathway and utilizes NF-kB, CEBP and AP-1 transcription factors. Sci. Rep..

[24] Ho M.H., Yen C.H., Hsieh T.H., Kao T.J., Chiu J.Y., Chiang Y.H., Hoffer B.J., Chang W.C., Chou S.Y. (2021). CCL5 via GPX1 activation protects hippocampal memory function after mild traumatic brain injury. Redox Biol..

[25] Rudemiller N.P., Patel M.B., Zhang J.D., Jeffs A.D., Karlovich N.S., Griffiths R., Kan M.J., Buckley A.F., Gunn M.D., Crowley S.D. (2016). C-C Motif Chemokine 5 Attenuates Angiotensin II-Dependent Kidney Injury by Limiting Renal Macrophage Infiltration. Am. J. Pathol..

[26] Marques R.E., Guabiraba R., Russo R.C., Teixeira M.M. (2013). Targeting CCL5 in inflammation. Expert. Opin. Ther. Targets..

[27] Grayson M.H., Holtzman M.J. (2006). Chemokine complexity: The case for CCL5. Am. J. Respir. Cell Mol. Biol..

[28] Pavlatou M.G., Papastamataki M., Apostolakou F., Papassotiriou I., Tentolouris N. (2009). FORT and FORD: Two simple and rapid assays in the evaluation of oxidative stress in patients with type 2 diabetes mellitus. Metabolism.

[29] Wu J., Yan L.J. (2015). Streptozotocin-induced type 1 diabetes in rodents as a model for studying mitochondrial mechanisms of diabetic β cell glucotoxicity. Diabetes Metab. Syndr. Obes..

[30] Rosholt M.N., King P.A., Horton E.S. (1994). High-fat diet reduces glucose transporter responses to both insulin and exercise. Am. J. Physiol..

[31] Hancock C.R., Han D.H., Chen M., Terada S., Yasuda T., Wright D.C., Holloszy J.O. (2008). High-fat diets cause insulin resistance despite an increase in muscle mitochondria. Proc. Natl. Acad. Sci. USA.

[32] Heath R.B., Karpe F., Milne R.W., Burdge G.C., Wootton S.A., Frayn K.N. (2007). Dietary fatty acids make a rapid and substantial contribution to VLDL-triacylglycerol in the fed state. Am. J. Physiol. Endocrinol. Metab..

[33] Miranda J., Eseberri I., Lasa A., Portillo M.P. (2018). Lipid metabolism in adipose tissue and liver from diet-induced obese rats: A comparison between Wistar and Sprague-Dawley strains. J. Physiol. Biochem..

[34] Johnson R.J., Lanaspa M.A., Gabriela Sánchez-Lozada L., Rodriguez-Iturbe B. (2015). The discovery of hypertension: Evolving views on the role of the kidneys, and current hot topics. Am. J. Physiol. Renal. Physiol..

[35] Susic D., Frohlich E.D. (2011). Hypertensive Cardiovascular and Renal Disease and Target Organ Damage: Lessons from Animal Models. Cardiorenal. Med..

[36] Coffman T.M. (2014). The inextricable role of the kidney in hypertension. J. Clin. Investig..

[37] Cuspidi C., Sala C., Zanchetti A. (2008). Metabolic syndrome and target organ damage: Role of blood pressure. Expert. Rev. Cardiovasc. Ther..

[38] Mulè G., Cerasola G. (2006). The metabolic syndrome and its relationship to hypertensive target organ damage. J. Clin. Hypertens..

[39] Dawood T., Schlaich M.P. (2009). Mediators of target organ damage in hypertension: Focus on obesity associated factors and inflammation. Minerva. Cardioangiol..

[40] Lorgis L., Zeller M., Dentan G., Sicard P., Richard C., Buffet P., L’Huillier I., Beer J.C., Cottin Y., Rochette L. (2010). The free oxygen radicals test (FORT) to assess circulating oxidative stress in patients with acute myocardial infarction. Atherosclerosis.

[41] Găman M.A., Epîngeac M.E., Diaconu C.C., Găman A.M. (2020). Evaluation of oxidative stress levels in obesity and diabetes by the free oxygen radical test and free oxygen radical defence assays and correlations with anthropometric and laboratory parameters. World J. Diabetes.

[42] Gizi A., Papassotiriou I., Apostolakou F., Lazaropoulou C., Papastamataki M., Kanavaki I., Kalotychou V., Goussetis E., Kattamis A., Rombos I. (2011). Assessment of oxidative stress in patients with sickle cell disease: The glutathione system and the oxidant-antioxidant status. Blood Cells. Mol. Dis..

[43] Aguilar Diaz De Leon J., Borges C.R. (2020). Evaluation of Oxidative Stress in Biological Samples Using the Thiobarbituric Acid Reactive Substances Assay. J. Vis. Exp..

[44] Cao G., Prior R.L. (1998). Comparison of different analytical methods for assessing total antioxidant capacity of human serum. Clin. Chem..

[45] Schlesier K., Harwat M., Böhm V., Bitsch R. (2002). Assessment of antioxidant activity by using different in vitro methods. Free Radic. Res..

[46] Moon J.K., Shibamoto T. (2009). Antioxidant assays for plant and food components. J. Agric. Food. Chem..

[47] Peng T.I., Jou M.J. (2010). Oxidative stress caused by mitochondrial calcium overload. Ann. N. Y. Acad. Sci..

[48] Monteiro R., Azevedo I. (2010). Chronic inflammation in obesity and the metabolic syndrome. Mediators. Inflamm..

[49] Yan L.J. (2014). Pathogenesis of chronic hyperglycemia: From reductive stress to oxidative stress. J. Diabetes Res..

[50] Vekic J., Stromsnes K., Mazzalai S., Zeljkovic A., Rizzo M., Gambini J. (2023). Oxidative Stress, Atherogenic Dyslipidemia, and Cardiovascular Risk. Biomedicines.

[51] Vasavada N., Agarwal R. (2005). Role of oxidative stress in diabetic nephropathy. Adv. Chronic. Kidney Dis..

[52] Ratliff B.B., Abdulmahdi W., Pawar R., Wolin M.S. (2016). Oxidant Mechanisms in Renal Injury and Disease. Antioxid. Redox Signal..

[53] Di Majo D., Sardo P., Giglia G., Di Liberto V., Zummo F.P., Zizzo M.G., Caldara G.F., Rappa F., Intili G., van Dijk R.M. (2022). Correlation of Metabolic Syndrome with Redox Homeostasis Biomarkers: Evidence from High-Fat Diet Model in Wistar Rats. Antioxidants.

[54] Maciejczyk M., Żebrowska E., Zalewska A., Chabowski A. (2018). Redox Balance, Antioxidant Defense, and Oxidative Damage in the Hypothalamus and Cerebral Cortex of Rats with High Fat Diet-Induced Insulin Resistance. Oxidative Med. Cell. Longev..

[55] Chang K.S., Chen S.T., Hsu S.Y., Sung H.C., Lin W.Y., Tsui K.H., Lin Y.H., Hou C.P., Juang H.H. (2024). The C-X-C Motif Chemokine Ligand 5, Which Exerts an Antioxidant Role by Inducing HO-1 Expression, Is C-X-C Motif Chemokine Receptor 2-Dependent in Human Prostate Stroma and Cancer Cells. Antioxidants.

[56] Lu S.C. (2013). Glutathione synthesis. Biochim. Biophys. Acta.

[57] Langston W., Circu M.L., Aw T.Y. (2008). Insulin stimulation of gamma-glutamylcysteine ligase catalytic subunit expression increases endothelial GSH during oxidative stress: Influence of low glucose. Free Radic. Biol. Med..

[58] Begleiter A., Fourie J. (2004). Induction of NQO1 in cancer cells. Methods Enzymol..

[59] Lee W.S., Ham W., Kim J. (2021). Roles of NAD(P)H:quinone Oxidoreductase 1 in Diverse Diseases. Life.

[60] Qiu D., Song S., Wang Y., Bian Y., Wu M., Wu H., Shi Y., Duan H. (2022). NAD(P)H: Quinone oxidoreductase 1 attenuates oxidative stress and apoptosis by regulating Sirt1 in diabetic nephropathy. J. Transl. Med..

[61] Yin F.F., Bailey S., Innis C.A., Ciubotaru M., Kamtekar S., Steitz T.A., Schatz D.G. (2009). Structure of the RAG1 nonamer binding domain with DNA reveals a dimer that mediates DNA synapsis. Nat. Struct. Mol. Biol..

[62] Steinbrenner H., Speckmann B., Klotz L.O. (2016). Selenoproteins: Antioxidant selenoenzymes and beyond. Arch. Biochem. Biophys..

[63] Barrett C.W., Short S.P., Williams C.S. (2017). Selenoproteins and oxidative stress-induced inflammatory tumorigenesis in the gut. Cell. Mol. Life Sci..

[64] Katsounas A., Schlaak J.F., Lempicki R.A. (2011). CCL5: A double-edged sword in host defense against the hepatitis C virus. Int. Rev. Immunol..

[65] Aldinucci D., Borghese C., Casagrande N. (2020). The CCL5/CCR5 axis in cancer progression. Cancers.

[66] Zeng Z., Lan T., Wei Y., Wei X. (2022). CCL5/CCR5 axis in human diseases and related treatments. Genes Dis..

[67] Ishida Y., Kimura A., Kuninaka Y., Inui M., Matsushima K., Mukaida N., Kondo T. (2012). Pivotal role of the CCL5/CCR5 interaction for recruitment of endothelial progenitor cells in mouse wound healing. J. Clin. Investig..

[68] Ho M.H., Tsai Y.J., Chen C.Y., Yang A., Burnouf T., Wang Y., Chiang Y.H., Hoffer B.J., Chou S.Y. (2024). CCL5 is essential for axonogenesis and neuronal restoration after brain injury. J. Biomed. Sci..

[69] Rudemiller N.P., Crowley S.D. (2017). The role of chemokines in hypertension and consequent target organ damage. Pharmacol. Res..

[70] Zhang Z., Wang Q., Yao J., Zhou X., Zhao J., Zhang X., Dong J., Liao L. (2020). Chemokine Receptor 5, a Double-Edged Sword in Metabolic Syndrome and Cardiovascular Disease. Front. Pharmacol..

[71] Kitade H., Sawamoto K., Nagashimada M., Inoue H., Yamamoto Y., Sai Y., Takamura T., Yamamoto H., Miyamoto K., Ginsberg H.N. (2012). CCR5 plays a critical role in obesity-induced adipose tissue inflammation and insulin resistance by regulating both macrophage recruitment and M1/M2 status. Diabetes.

[72] Kester M.I., van der Flier W.M., Visser A., Blankenstein M.A., Scheltens P., Oudejans C.B. (2011). Decreased mRNA expression of CCL5 [RANTES] in Alzheimer’s disease blood samples. Clin. Chem. Lab. Med..

[73] Bouabout G., Ayme-Dietrich E., Jacob H., Champy M.F., Birling M.C., Pavlovic G., Madeira L., Fertak L.E., Petit-Demoulière B., Sorg T. (2018). Nox4 genetic inhibition in experimental hypertension and metabolic syndrome. Arch. Cardiovasc. Dis..

[74] Nunes-Souza V., César-Gomes C.J., Da Fonseca L.J., Guedes Gda S., Smaniotto S., Rabelo L.A. (2016). Aging Increases Susceptibility to High Fat Diet-Induced Metabolic Syndrome in C57BL/6 Mice: Improvement in Glycemic and Lipid Profile after Antioxidant Therapy. Oxidative Med. Cell. Longev..

[75] Alquoqa R.S., Kasabri V., Naffa R., Akour A., Bustanji Y. (2018). Cross-sectional correlates of myeloperoxidase and alpha-1-acid glycoprotein with adiposity, atherogenic and hematological indices in metabolic syndrome patients with or without diabetes. Ther. Adv. Endocrinol. Metab..

