# Supplementary material for: Transcriptomic Redox Dysregulation in a Rat Model of Metabolic Syndrome-Associated Kidney Injury
Source: Antioxidants (Basel). 2025 Jun 17;14(6):746. doi: 10.3390/antiox14060746 (PMC12189155; doi:10.3390/antiox14060746)
Supplement: Supplementary file 1 [file antioxidants-14-00746-s001.zip › antioxidants-3671219-supplementary.pdf]

Transcriptomic Redox Dysregulation in a Rat Model of Metabolic Syndrome-Associated Kidney Injury

Chien-Lin Lu[1,2,] Yi-Yun Wang[3], Yih-Jeng Tsai[1,4], Hsuan-Ting Chen[1], Ming-Chieh Ma[1*] and Wen-Bin Wu[1,3*]

[1]School of Medicine, College of Medicine, Fu Jen Catholic University, New Taipei City 242062, Taiwan.

[2]Division of Nephrology, Department of Internal Medicine, Fu Jen Catholic University Hospital, Fu Jen Catholic University, New Taipei City 243089, Taiwan.

[3]Graduate Institute of Biomedical and Pharmaceutical Science, Fu Jen Catholic University, New Taipei City, Taiwan.

[4]Department of Otolaryngology Head and Neck Surgery, Shin Kong Wu Ho-Su Memorial Hospital, Taipei, Taiwan.

*Ming-Chieh Ma and Wen-Bin Wu equally contributed to this work for correspondence.

Supplementary Figure and Table

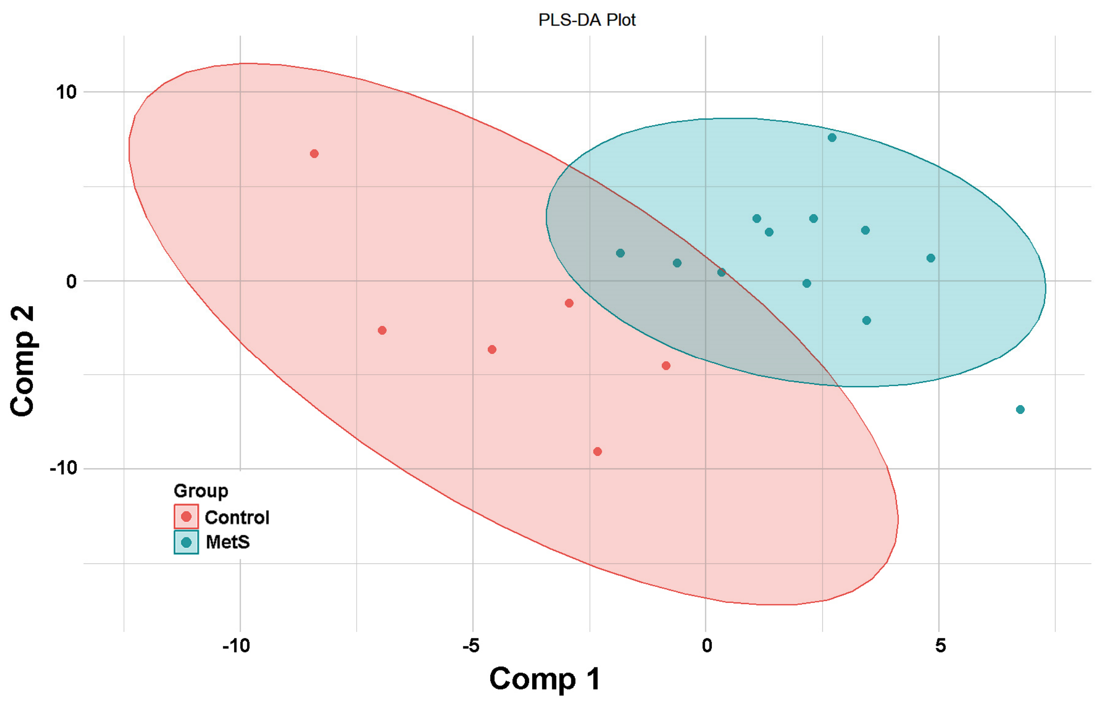

Figure S1. Partial least squares-discriminant analysis (PLS-DA). Cross-validated PLS-DA score plot comparing redox gene expression profiles between the control group (red dots, n = 6) and the

metabolic syndrome (MetS) group (green dots, n = 12), showing clear separation based on multivariate gene expression patterns. The analysis was performed using R software (version 4.3.3, The R Project for Statistical Computing). PLS-DA was conducted using the "pls" package (version 2.8-5), which includes functions for Partial Least Squares Regression (PLSR) and Principal Component Regression. The PLSR function was applied to construct models based on ΔCt-transformed gene expression data. Model performance was evaluated by extracting component scores and loadings using the "scores" function. Colored ellipses represent 95% confidence intervals. Components (comp) 1 and 2 accounted for 11.1% and 14.0% of the total variance, respectively.

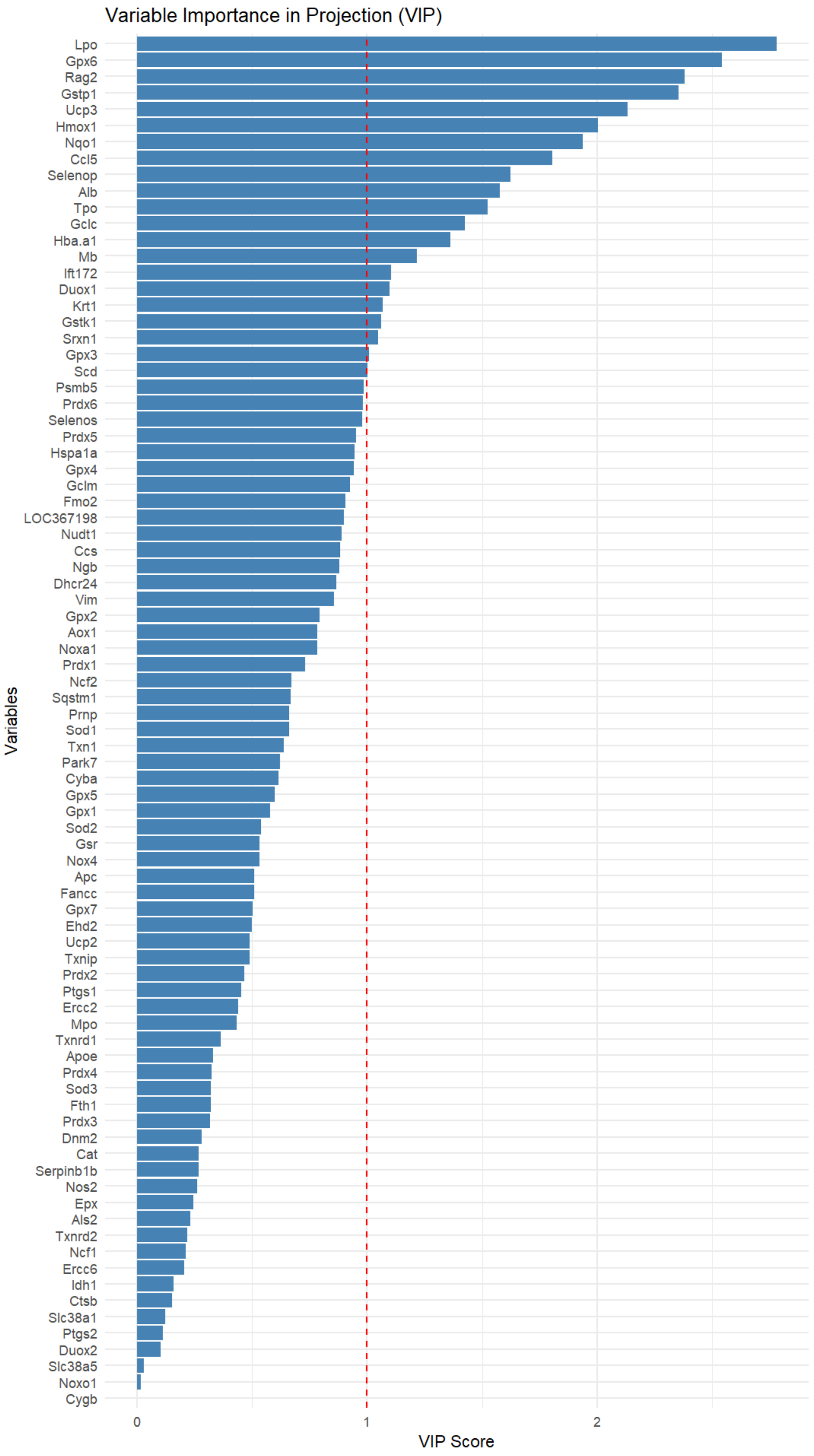

Figure S2. Variable Importance in Projection (VIP) Score Plot. VIP scores were derived from the PLS-DA model shown in Figure S1. To identify key variables contributing to group separation, VIP values were calculated using the VIP function. Genes with a VIP score greater than 1.0 were considered significant discriminators between control and MetS groups. Notably, CCL5 exhibited

a VIP score of 1.8062, ranking among the top contributors, thereby supporting its biological relevance in the context of metabolic syndrome.

Table S1. List of VIP scores from PLS-DA model for six downregulated genes. The six downregulated genes are listed from high to low VIP scores. This highlights CCL5's potential role in transcriptomic shifts associated with metabolic syndrome. Given its controversial roles in some diseases and metabolic dysfunction, CCL5 was selected for downstream validation by qPCR.

Gene	VIP Score
Gpx6	2.5409
Rag2	2.3807
NQO1	1.9373
CCL5	1.8062
SEPP1	1.6224
Gclc	1.4268
